# MYCN in Neuroblastoma: “Old Wine into New Wineskins”

**DOI:** 10.3390/diseases9040078

**Published:** 2021-10-29

**Authors:** Maria Braoudaki, Kyriaki Hatziagapiou, Apostolos Zaravinos, George I. Lambrou

**Affiliations:** 1 Department of Life and Environmental Sciences, School of Life and Health Sciences, University of Hertfordshire, Hatfield AL10 9AB, Hertfordshire, UK; m.braoudaki@herts.ac.uk; 2 Choremeio Research Laboratory, First Department of Pediatrics, National and Kapodistrian University of Athens, Thivon & Levadeias 8, Goudi, 11527 Athens, Greece; khatziag@med.uoa.gr; 3 Department of Life Sciences, European University Cyprus, Diogenis Str., 6, Nicosia 2404, Cyprus; 4 Cancer Genetics, Genomics and Systems Biology Group, Basic and Translational Cancer Research Center, European University Cyprus, Nicosia 1516, Cyprus

**Keywords:** MYCN, amplification, epigenetic regulation, acetylation, G-quadraplex, neuroblastoma

## Abstract

MYCN Proto-Oncogene, BHLH Transcription Factor (MYCN) has been one of the most studied genes in neuroblastoma. It is known for its oncogenetic mechanisms, as well as its role in the prognosis of the disease and it is considered one of the prominent targets for neuroblastoma therapy. In the present work, we attempted to review the literature, on the relation between MYCN and neuroblastoma from all possible mechanistic sites. We have searched the literature for the role of MYCN in neuroblastoma based on the following topics: the references of MYCN in the literature, the gene’s anatomy, along with its transcripts, the protein’s anatomy, the epigenetic mechanisms regulating MYCN expression and function, as well as MYCN amplification. *MYCN* plays a significant role in neuroblastoma biology. Its functions and properties range from the forming of G-quadraplexes, to the interaction with miRNAs, as well as the regulation of gene methylation and histone acetylation and deacetylation. Although *MYCN* is one of the most primary genes studied in neuroblastoma, there is still a lot to be learned. Our knowledge on the exact mechanisms of MYCN amplification, etiology and potential interventions is still limited. The knowledge on the molecular mechanisms of MYCN in neuroblastoma, could have potential prognostic and therapeutic advantages.

## 1. Introduction

### 1.1. A Brief “Ensemble” to MYCN

*MYCN* is one of the most studied genes with respect to neuroblastoma. This gene was identified back in 1983 by Kohl et al. (1983) [1] and Schwab et al. (1983) [2], which was initially found to be amplified in neuroblastoma cases, “homologous to *v-myc* but different from *MYC* in human neuroblastoma” [3]. Later on, *MYCN* was found to manifest high expression levels, as a result of gene amplification, in neuroblastoma cell lines, metastatic neuroblastoma, retinoblastoma and lung tumors [4]. The first report on *MYCN* (as previously was named as *myc*, *n-myc*), was by Schwab and Bishop (1988), where they have reported that besides its role in human tumors, it participated in various biological processes, including senescence [5], resistance to therapy [6] and most interestingly it was found that “circular extra-chromosomal DNA molecules could transport amplified MYCN proto-oncogenes in human neuroblastomas” [7].

*MYCN* has been extensively studied both in fetal development, as well as for its role in human neoplasms. *MYCN* products are nuclear phosphoproteins with a short half-life. It has been shown that in terms of its physiological cellular function, it plays a role in neural development of the sympathetic system. Especially, the expression of *MYCN* increases significantly during the fetal stages. It has been shown, in multiple reports, that *MYCN* overexpression is directly associated with poor prognosis of neuroblastoma and it is thought that there is a strong correlation between gene overexpression and the evolutionary course of tumors [8]. On the other hand, *MYCN* has received less attention with respect to pediatric brain tumors, while recent reports have highlighted its role in the disease [9,10,11]. Apart from its role in neuroblastoma, *MYCN* has been studied in some extent for other pediatric brain neoplasms such as medulloblastoma [12,13], astrocytoma [14], glioblastoma [15,16], and others. One of the main questions in MYCN biology is the link between its overexpression and amplification. The gene’s overexpression does not always correlate to the gene’s copy numbers and this is a topic under continuing investigation.

The *MYCN* oncogene is one of the most important genetic biomarkers for the diagnosis, prognosis and treatment of neuroblastoma. *MYCN* overexpression is associated with poor prognosis and rapid tumor growth [9,17,18]. However, it does not function as a unique f actor rather it is reported to participate in a network of other factors, procuring neuroblastoma’s pathology. Examples of other genes linked to MYCN biology is the *ID2* gene, which is also linked to *MYCN* overexpression and the fast development of the disease as studies have demonstrated the ability of MYCN to act as a transcription factor for ID2 [19]. Similar case is the NF2 transcription factor, whose methylation is linked to neuroblastoma progression [20]. One significant player in tumor progression, TP73 tumor suppressor gene, could not be left out of tumor progression. MYCN overexpression and TP53 downregulation is considered (and actually very recently) as a twin-target for neuroblastoma therapy [21], but also is linked to advanced stages of the disease.

Beside the aforementioned gene aberrations, chromosomal aberrations are also known to play a significant role in neuroblastoma progression. For example, deletion of the 1p36 region is also associated with poor disease prognosis, and the region has not yet been associated with a tumor suppressor gene [22,23]. Further studies, lead to the conclusion that the chromosomal region 1p36 contains some tumor suppressor genes which, however, have not yet been detected. Another negative prognostic indicator for the poor progression of the disease is the unbalanced enhancement of the chromosomal region 17q. The expression of TRKA, TRKB and TRKC receptors (neurotrophic tyrosine kinase receptors Types 1, 2, 3) has been associated with a good prognosis of the disease, while the gene appears to be suppressed in cases where we have *MYCN* amplification [24]. Other chromosomal aberrations are also known to play a significant role in neuroblastoma progression such as 17q and 22p [25]. These chromosomal regions are known to code for tumor suppressor genes, which when deleted lead to disease progression.

### 1.2. The Frequency of MYCN in the Literature

**Figure 1:**
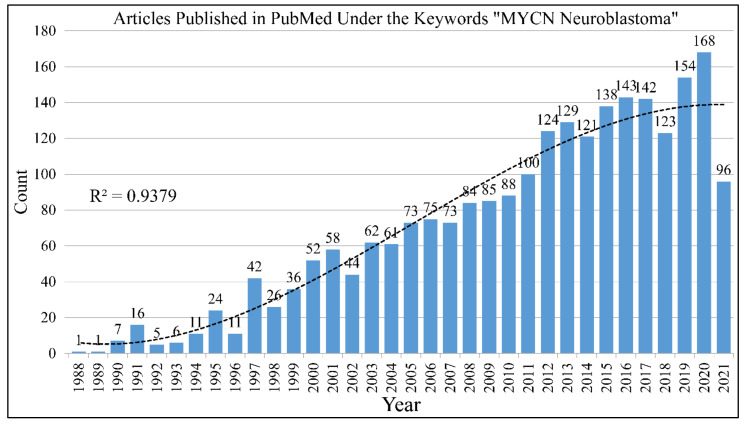
The literature concerning the role of MYCN in neuroblastoma. A total of 2166 citations come up in the PubMed database with the keywords “MYCN” and “neuroblastoma”.

The search with the keywords “MYCN Neuroblastoma” in PubMed returns 2166 results until June 12th 2021 (Figure 1). The gene, is one of the most studied genes for a specific disease. Interest on the MYCN gene and neuroblastoma biology remained constant throughout the years. In its almost 40 year history since its first reports, a lot is still to be learned on its role in neuroblastoma. The study of MYCN for other types of tumors returns much less results, as for example the keywords “MYCN brain tumor” returns 237 hits until 12 June 2021.

### 1.3. A Brief Reference (and History) to Neuroblastoma

Neuroblastoma was discovered in 1910 by Wright, JH (1910), where in his work he referred to the tumor as “neurocytoma” or “neuroblastoma”. In particular, he referred to neuroblastoma as “*The essential cells of the tumor are considered to be more or less undifferentiated nerve cells or neurocytes or neuroblasts, and hence the names neurocytoma and neuroblastoma*” [26]. Knowledge on the nature and biology of neuroblastoma increased drastically since that time. Prognosis and therapy have greatly improves although in many cases neuroblastoma is still fatal.

Neuroblastomas afford the most common extracranial solid tumors of childhood [27]. They commonly occur during the first five years of life and may arise during infancy. Potential localizations include the sympathetic nervous system and occasionally the brain, but they are also common in the abdomen; most cases arise in either the adrenal medulla or the peritoneal sympathetic ganglia. Ganglion cell tumors, neuroblastoma and ganglioneuroma, are more common mainly in childhood, but their clinical manifestation vary.

These neoplasms occur most commonly during the first five years of life and may arise during infancy. Most neuroblastomas are sporadic, although familial cases occur as well. Neuroblastomas may occur anywhere in the sympathetic nervous system and occasionally within the brain, but they are also common in the abdomen; most cases arise in either the adrenal medulla or the peritoneal sympathetic ganglia. The adrenal medulla derives from the neural crest. It contains two major cell types chromaffin and ganglion cells. Chromaffin cells (pheochromocytes) are the predominant medullary cells. They are postganglionic sympathetic neurons that have lost their axons and dendrites. They synthesize and release their catecholamines (epinephrine or norepinephrine) in response to neural stimulation (especially stress) mediated by preganglionic sympathetic neurons. The few parasympathetic ganglion cells present exhibit typical morphological characteristics of autonomic ganglion cells.

Under normal circumstances, as aforementioned, chromaffin cells produce two types of catecholamines in response to pregaglionic sympathetic stimulation (e.g., stress). Both elevate blood glucose by stimulating hepatic glycogenolysis. They also increase blood flow to the heart. Epinephrine increases the heart rate and dilates blood vessels to the organs needed to escape stress, such as cardiac and skeletal muscles. It dilates bronchioles and constricts the vessels in organs (e.g., skin, digestive tract, kidneys) that are not essentials in reacting to stress. Norepinephrine also constricts blood vessels in non-essential organs increasing peripheral resistance and as a result it increases blood pressure and blood flow to the heart, brain and skeletal muscles.

Under abnormal function, hypersecreting chromaffin cell tumors (pheochromocytomas) cause a sustained stress response (especially hypertension) even in the absence of stress. While neuroblastoma is a highly malignant tumor of early life (related by maturation to ganglioneuroma), the other principal tumor of the sympathetic nervous system, pheochromocytoma, is usually a benign tumor of adults, unrelated to either neuroblastoma or ganglioneuroma [28]. Ganglion cell tumors, neuroblastoma and ganglioneuroma, are more common, mainly in childhood, but their clinical manifestation vary.

#### 1.3.1. Clinical Characteristics of Neuroblastoma

Clinical features are extremely variable and reflect the widespread distribution of neural crest tissue. Metastases are common at diagnosis, approximately half of newly diagnosed patients have distant metastases, and often cause the symptoms that lead to the diagnosis of neuroblastoma. These symptoms are divided into specific and nonspecific. Remote effects are occasionally seen. There are three most widely used neuroblastoma staging systems: Evans, Pediatric Oncology Group (POG) and TNM- Union Internationale Contre le Cancer (UICC) [29].

Tumor markers are extremely useful in evaluating children who have neuroblastoma. Concerning urinary markers, catecholamines, are elaborated by most tumors are useful for diagnosis, follow up on response to therapy, and detection of recurrence. On the other hand, serum markers are used, whom elevations are often associated with a poor prognosis. Such markers are ferritin and lactate dehydrogenase. Last but not least, MYCN represents an oncogene marker whose amplification within the tumor cells is also associated with poor prognosis [30]. MYCN oncogene is a member of myc family of oncogenes that encode transcription factors regulating cell growth. The additional copies of this oncogene contribute to the rapid growth of the neuroblastomas that have MYCN amplification and thus poor prognosis [31]. Prognosis depends on the following factors: age, stage, histopathology (degree of differentiation of tumor cells and their pattern of growth) and tumor markers [32].

Therapy includes surgery, aggressive multi-agent chemotherapy treatment, especially if the patient has poor prognostic features such as MYCN amplification and autologous bone marrow transplantation according to the stage diagnosed. In addition, spontaneous regression without any therapy is common in a particular stage [33].

#### 1.3.2. Oncogenetic Mechanisms in Neuroblastoma

Cell cycle is divided in four phases, designated G1, S (when DNA synthesis occurs), G2 and M (mitosis, when the cell actually divides). Cells that have ceased dividing (permanently or temporarily) are said to be in a resting phase called G0. The ordered progression from one phase to the next is coordinated by a complex set of proteins, and the cellular signals that control cell division do so by modulating the activities of those proteins which include cyclins, CDKs (cyclin-dependent kinases), CDKIs (CDK inhibitors) [34]. Progression through G1, is also marked by the accumulation of other transcription factors, which presumably act on other genes required for DNA replication. Several of these factors are regulated by association with other proteins.

The ultimate stage of the signal transduction pathway is regulation of DNA transcription in the nucleus. Components of the signal transduction factors that regulate the activity of specific genes whose proteins products influence cellular growth and proliferation. Genes that encode these transcription factors include Myc, Fos and Jun. Mutations may occur in any of these steps involved in regulation of cell growth and differentiation. Accumulation of such mutations within a cell lineage may result in progressive deregulation of growth eventually producing a tumor cell. Proto-oncogenes encode products that control cell growth and differentiation. When mutated, they may become oncogenes, which can cause cancer. Most oncogenes act as dominant gain-of-function mutations that lead to deregulation of cell cycle control [35]. In contrast to tumor suppressor genes, most oncogenes do not exhibit germline mutations that cause inherited cancer syndromes [36]. Instead, somatic mutations are observed that lead to sporadic cancers.

Retroviruses are a type of RNA virus that is capable of using reverse transcriptase to transcribe RNA into DNA. In this way, the RNA genome of the retrovirus is converted to DNA, which can be inserted into a chromosome of a host cell. In an earlier cycle of infection, a retrovirus may have incorporated in a mutant oncogene from the genome of the host. When the retrovirus invades a new cell, it can transfer the oncogene in the genome of the new host, thus transforming the cell. Transforming retroviruses have also identified the nuclear transcription factor genes Myc, Jun and Fos, as other molecular components capable of initiating cell transformation. The transcription factor gene associated with neuroblastoma is the oncogene MYCN that encodes a DNA-binding protein and is located on the 2p24 chromosome [37]. A recent study showed that in neuroblastoma the human endogenous retroviruses was abundant, indicating a possible role in the ontogenesis of the disease [38]. Interestingly, another recent report has shown that enterovirii responsible for other conditions, are implicated in the nerve tissue and possibly in tumorigenesis, including neuroblastoma [39]. The role of viral genome in neuroblastoma is still under investigation and consists of an active research field.

Myc protein represents a specific example of HLH (helix–loop–helix) proteins which consist of short alpha helix connected by a loop to a longer alpha helix. The loop allows dimerization of two HLH proteins to occur and form a Y-shaped dimer. Dimerization may occur between two of the same proteins (homodimers) or two different proteins (heterodimers) [40].

Differences in the level of expression of encoded proteins can, among others, cause proto-oncogenes to become oncogenic. In childhood neuroblastoma, the amount of the MYCN gene is increased from a diploid number to many dozens per cell. MYCN amplification is associated with poor prognosis especially in older children than infants and is a significant factor in the aggressiveness of the tumor [41].

An older DNA microarray analysis suggested there is a link between DNA methylation and MYCN gene expression, i.e., there is evidence DNA methylation may directly control MYCN oncoprotein levels [42]. Epigenetic silencing of potential tumor suppressor genes as an alternative mechanism in the absence of genetic mutations has not yet been studied systematically in neuroblastomas and needs further investigation.

In contrast to *MYCN* gene amplification, the degree of expression of the *MYCN* gene in the tumor does not predict prognosis [43,44], but it has been previously shown that the MYCN product (MYCN) is a nuclear phosphoprotein, which can transcriptionally activate many genes, either directly (e.g., ID2) or indirectly [45]. In childhood neuroblastoma, the amount of the MYCN gene is increased from a diploid number to many dozens per cell. MYCN amplification has been associated with poor prognosis especially in older children when compared to infants, while it affords a significant factor for tumor aggressiveness [41].

#### 1.3.3. Prognosis of Neuroblastoma

Since the advent of *MYCN* discovery, it is directly linked to the disease’s prognosis. Up-to-date, several factors have been added to the criteria for neuroblastoma’s prognosis. A recent study highlighted that exportin-T overexpression, is an independent poor prognosis factor in *MYCN* amplified neuroblastoma [46]. Further on, the most recent neuroblastoma risk classification system indicated that the presence of image defined risk factors (IDRF), MYCN amplification and age are important prognostic factors related to poor prognosis [47]. Another prognostic factor recently mentioned was the overexpression of c-myc, which was confirmed to be linked to poor prognosis independently of *MYCN* amplification [48]. Further on, in *MYCN* non-amplified neuroblastoma, the *DST* gene was linked to good prognosis, with patients overexpressing DST manifesting higher survival rates [49]. In cases of metastatic neuroblastoma (those with bone marrow metastasis) aberrations in chromosome 10, along with *MYCN* amplification [22]. Finally, an interesting recent study indicated the role of the microenvironment in neuroblastoma prognosis. In particular, it has been reported that low expression of IL5 and NKT in the tumor neuroblastoma, was linked to poor outcome in MYCN non-amplified tumors [50].

### 1.4. Scope of the Present Work

In the present work, we review the literature for the significance of MYCN, in neuroblastoma. We present old and new knowledge on the prognostic, diagnostic and therapeutic properties of MYCN in neuroblastoma.

## 2. The MYCN Gene

The *Myc* genes consist of a family of regulator genes and proto-oncogenes whose products are transcription factors. The *Myc* family consists of “three related human genes: *c-myc* (*MYC*), *l-myc* (*MYCL*), and *n-myc* (*MYCN*)”. *MYC* was the first gene of this family to be discovered. Its name was derived from the *v-myc* gene, which was the first gene to be discovered in this family. The term c-myc, was given due to the homology of *c-myc* to the *v-myc* gene, which is of viral origin. The *v-myc* gene is an oncogene present in the avian myelocytomatosis virus, as well as a human oncogene upregulated in various tumors. Following the discovery of *c-myc* other members of this family were discovered and named *n-Myc* and *l-Myc* [51].

### 2.1. The “Anatomy” of MYCN

**Figure 2:**
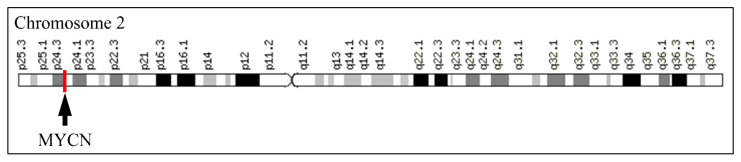
The location of *MYCN* on Chromosome 2.

**Figure 3:**
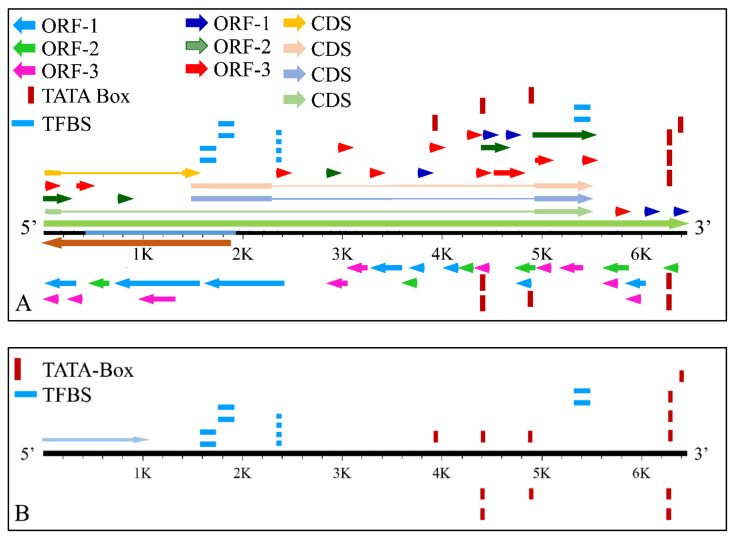
The analysis of the *MYCN* gene with its complete anatomy (**A**) and the TATA boxes (**B**) (ORF: open reading frame, TFBS: transcription factor binding site, CDS: gene coding region).

The *MYCN*, is constructed by 6455 nucleotides and is located on Chromosome 2 with exact location 2p24.3 and has three exons (Figure 2). It can be accessed through the gene ID 4613 (https://www.ncbi.nlm.nih.gov/gene/4613, accessed on 18 June 2021) in the NCBI, Gene database with official name is “*MYCN* proto-oncogene, bHLH transcription factor”. The complete anatomy of the gene is presented in Figure 3A and Table 1. The gene has twelve TATA-Box sites, which are located between nucleotides 3930 and 6382 (Figure 3B and Table 2).

#### 2.1.1. Transcription Factor Binding Sites

Gene regulation is further controlled by transcription factors, on the so-called transcription factor binding sites (TFBS). TFBS can be predicted either experimentally or bioinformatically. In the present work we have attempted to identify possible TFBS on the MYCN gene using the *Webgestalt* (http://www.webgestalt.org/, accessed on 20 June 2021) web-based tool [52,53,54,55]. Hence, predicted TFBS were the NF1, E2F, AP4 and FREAC2. Further on, two regions on the gene’s product were identified as potential interacting sites with AURKA and FBXW7 [56], and one site was predicted to be a Leucine-zipper region.

#### 2.1.2. Gene Ontology (GO) Annotation

The gene ontology annotation for the gene reports as functions: “enables DNA binding” [57], “enables DNA-binding transcription activator activity” [58], “RNA polymerase II-specific” [59], “enables DNA-binding transcription factor activity” [60], “enables DNA-binding transcription factor activity, RNA polymerase II-specific” [60], “enables RNA polymerase II cis-regulatory region sequence-specific DNA binding” [58], “enables kinase binding” [56], “enables protein binding” [56,57,61,62,63,64,65,66,67,68,69] and “enables protein dimerization activity” [70]. In addition, the gene’s biological processes are: “involved in branching morphogenesis of an epithelial tube”, “involved in cartilage condensation”, “involved in embryonic digit morphogenesis”, “involved in embryonic skeletal system morphogenesis”, “involved in lung development”, “involved in negative regulation of astrocyte differentiation”, “involved in negative regulation of gene expression” [71], “involved in negative regulation of reactive oxygen species metabolic process”, “involved in positive regulation of cell death”, “involved in positive regulation of gene expression” [72], “involved in positive regulation of mesenchymal cell proliferation”, “involved in positive regulation of production of miRNAs involved in gene silencing by miRNA” [71], “involved in positive regulation of transcription by RNA polymerase II” [58], “involved in positive regulation of transcription, DNA-templated” [73], “involved in regulation of inner ear auditory receptor cell differentiation”, “involved in regulation of transcription by RNA polymerase II” [60] and “involved in regulation of transcription by RNA polymerase II” [59].

#### 2.1.3. G-Quadraplexes in MYCN

**Figure 4:**
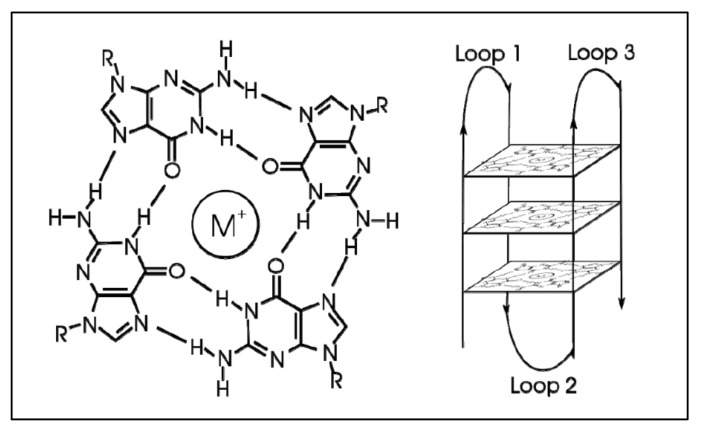
G-Quadraplex. A tetrad forms with the addition of an ion (left), while multiple tetrads form the quadraplex (https://en.wikipedia.org/wiki/G-quadruplex#cite_note-ReferenceC-4, accessed on 20 June 2021).

G-quadraplexes were first discovered in the early 1960s, where four-stranded DNA structures were discovered in nucleotide sequences rich in guanines [74]. G-quadruplex are tertiary DNA structures, which are formed in nucleic acid polymers that are rich in guanine [75] (Figure 4). G-quadraplexes can take different shapes, where each one contains “guanine-tetrads”, forming single- [76], double- [77], and quadruple-strands [78]. G-quadraplexes usually form in the telomeric regions, yet they form on oncogene sites [79]. The mechanism through which such a quadruplex forms is known to be through the Hoogsteen hydrogen bonding, creating a square planar or also known as “guanine-tetrad”. When multiple tetrads assemble or “stack” form the G-quadraplex. There are several variations of G-quadraplexes. These different forms can be divided into the intramolecular and intermolecular complexes. The intramolecular complexes are further divided to the parallel, anti-parallel, hybrid and higher order complexes, while the intermolecular complexes are further divided to bimolecular, trimolecular and tetramolecular structures [80]. The quadraplex is stabilized by the presence of a cation, which in most cases is potassium, yet other cations can also participate in quadruplex stabilization. G-quadraplexes can be predicted computationally and even experimentally through crystallization studies.

**Figure 5:**
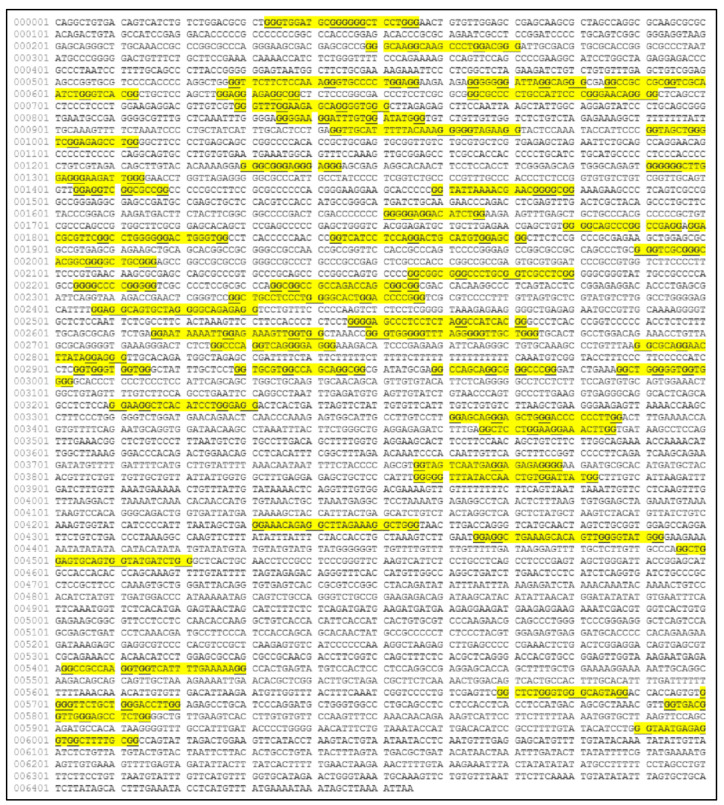
The predicted G-quadraplex sequences on the *MYCN* nucleotide sequence (image obtained from the QGRS Mapper web-tool, https://bioinformatics.ramapo.edu/QGRS/index.php, accessed on 21 June 2021. Map was reconstructed using the gene ID 4613 from the NCBI site).

**Figure 6:**
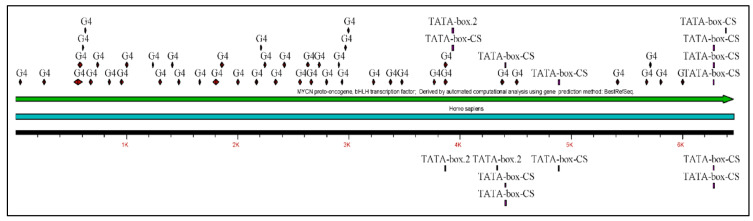
The reconstructed *MYCN* map based on the previous predictions from Figure 5 (legend: G4: G-Quadraplex position).

**Figure 7:**
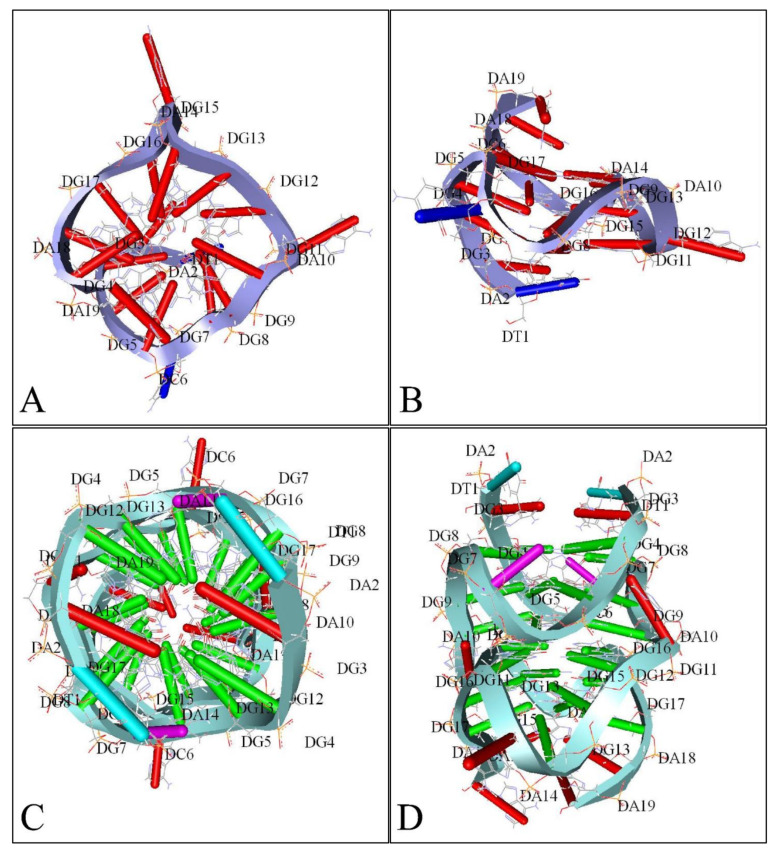
A 3D reconstruction of *MYCN* G-Quadraplexes. The transversal (**A**) and lateral (**B**) views of the 5′-T-A-G-G-G-C-G-G-G-A-G-G-G-A-G-G-G-A-A-3′nucleotide sequence’s G-quadraplex are presented as depicted from the deposited structure 2LEE (https://www.rcsb.org/structure/2LEE, accessed on 21 June 2021) in the Protein Data Bank (PDB) database (https://www.rcsb.org/, accessed on 21 June 2021) [82]. Similarly, The transversal (**C**) and lateral (**D**) views of the 5′-T-A-G-G-G-C-G-G-G-A-G-G-G-A-G-G-G-A-A-T-A-G-G-G-C-G-G-G-A-G-G-G-A-G-G-G-A-A-3′ nucleotide sequence’s G-quadraplex are presented as depicted from the deposited structure 2LED (https://www.rcsb.org/structure/2LED, accessed on 21 June 2021) in the PDB [82] (available structures have been reproduced with the Accelrys Discovery Studio software v.2.5).

Similarly, several studies (but not many) have investigated the presence of G-quadraplexes in the MYCN gene. We have also investigated the possibility of the presence of G-quadraplexes on the MYCN gene, using the QGRS Mapper [81]. In Figure 5 we provide an overview of the possible sites, where G-quadraplexes can form based on the nucleotide sequence as well as in Figure 6, we have designed a map of G-Quadraplexes on the gene itself. Interestingly, further studies have discovered the 3D structure of MYCN G-quadraplexes. One recent study, has reported on the potential G-quadraplex structures of MYCN (Figure 7), where they have investigated the nucleotide sequences 5′-T-A-G-G-G-C-G-G-G-A-G-G-G-A-G-G-G-A-A-3′ (Figure 7A,B) as well as the nucleotide sequence 5′-T-A-G-G-G-C-G-G-G-A-G-G-G-A-G-G-G-A-A-T-A-G-G-G-C-G-G-G-A-G-G-G-A-G-G-G-A-A-3′ (Figure 7C,D).

The presence of G-quadraplexes in the MYCN gene, plays a functional role, where it has been shown that they are very stable at the promoter site, regulating the gene’s expression [83]. The importance of MYCN G-quadraplexes, with respect to its prognostic, diagnostic and therapeutic use is still under investigation. However, some studies have highlighted that chemotherapeutics are able to recognize G-quadraplexes as for example, the alkaloids tetrandrine and isotetrandrine were able to bind to MYCN G-quadraplex [84]. From the same study, it was shown that tetrandrine had a “high possibility of binding to the MYCN G-quadraplexes” through hydrogen bonding, while isotetrandrine did not manifest the same affinity. However, it appeared that G-quadraplexes could be possible attractive sites for tumor therapy [84]. Similarly, another study showed that Enniatin B, “a well-known antibacterial, antihelmintic, antifungal, herbicidal, and insecticidal compound” [85], manifested high binding affinity to MYCN G-quadraplex implying possible therapeutic effects [86].

### 2.2. The “Anatomy” of MYCN’s Transcripts

The MYCN gene transcribes to four known transcripts namely the *MYCN* proto-oncogene, bHLH transcription factor (MYCN), Transcript Variant 1, mRNA (NM_001293228.2, accessed on 20 June 2021), the *MYCN* proto-oncogene, bHLH transcription factor (MYCN), Transcript Variant 3, mRNA (NM_001293231.2, accessed on 20 June 2021), the *MYCN* proto-oncogene, bHLH transcription factor (MYCN), Transcript Variant 2, mRNA (NM_001293233.2, accessed on 20 June 2021) and the *MYCN* proto-oncogene, bHLH transcription factor (MYCN), Transcript Variant 2, mRNA (NM_005378.6, accessed on 20 June 2021).

#### 2.2.1. MYCN Proto-Oncogene, bHLH Transcription Factor (MYCN), Transcript Variant 1, mRNA

**Figure 8:**
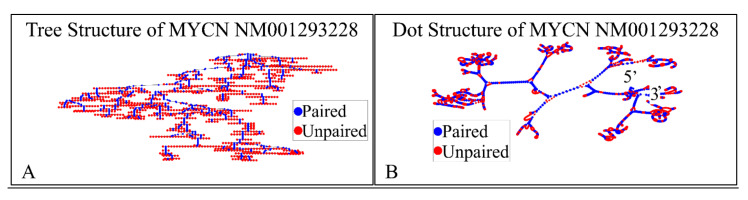
The predicted secondary structure of the *MYCN*, NM001293228 transcript. In particular, the tree structure (**A**) and the dot structure (**B**) of the transcript are presented (predictions were performed with the Matlab^®^ (The Mathworks, Inc., Natick, MA, USA), using the RNAfold method).

The NM001293228 mRNA, is a 2923 nucleotide long transcript. It consists of three exons, where the first ranges from Nucleotide 1 to 504, the second from Nucleotide 505 to 1411 and the third from Nucleotide 1412 to 2923. The gene coding region is from Nucleotide 622 to 2016. We have also calculated the secondary structure of the transcript, which is shown in Figure 8. There is not known how the secondary structure of this transcript participated in neuroblastoma progression and ontogenesis.

**Figure 9:**
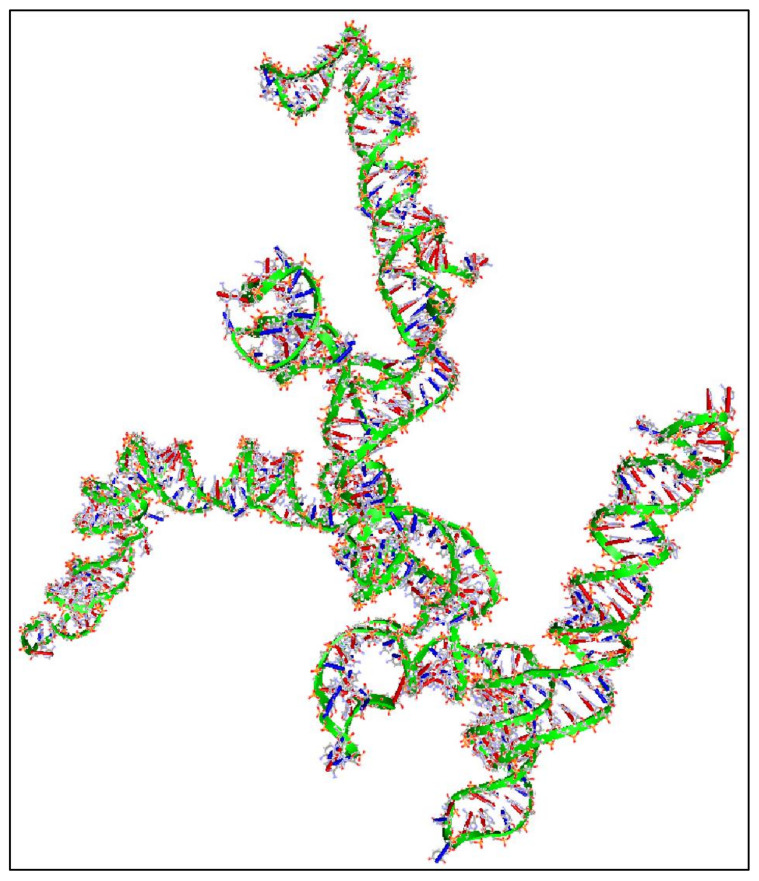
The predicted 3D structure of the *MYCN*, NM001293228 transcript and in particular, of the first exon (Nucleotides 1–504) (3D structure was predicted using the RNAcomposer web-tool [87,88] and it was then visualized using the Accelrys Discovery Studio).

In addition, we have calculated the 3D structure of the first exon of this specific transcript (Nucleotides 1–504). The reason, why we have tested the first 500 nucleotides, was due to the fact that it is computationally very challenging to create 3D predictions of large molecules. Just for reference, the secondary structure prediction of the ~3000 long nucleotide, needed approximately 15 h of computation in i7 8-core computer with 24 GB memory capacity and two 1GB CUDA graphics cards. This points out, not only the difficulty of calculating and predicting the 3D structures of RNAs, but also, and even more, the prediction of their functional properties. Out of pure curiosity, we wanted to examine and visualize how a 3D mRNA would look, and in addition, if we could find something about its functional properties (Figure 9). There are no known properties for the functional role of the 3D MYCN’s RNA structure, which makes it an interesting topic for future research.

#### 2.2.2. MYCN Proto-Oncogene, bHLH Transcription Factor (MYCN), Transcript Variant 3, mRNA

**Figure 10:**
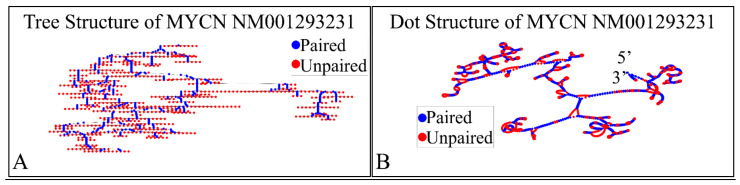
The predicted secondary structure of the *MYCN*, NM001293231 transcript. In particular, the tree structure (**A**) and the dot structure (**B**) of the transcript are presented (predictions were performed with the Matlab^®^ (The Mathworks, Inc. Natick, MA, USA), using the RNAfold method).

**Figure 11:**
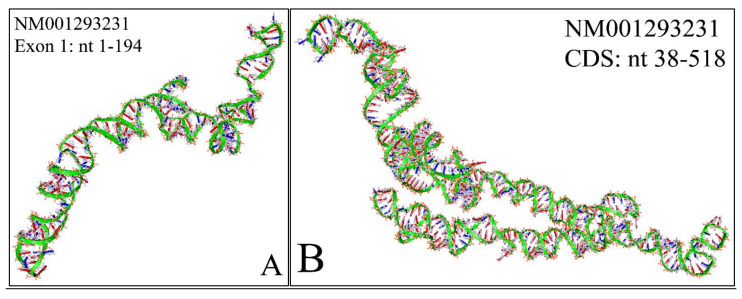
The predicted 3D structure of the *MYCN*, NM001293231 transcript and in particular, of the first exon (Nucleotides 1–194) (**A**) and the CDS (Coding DNA Sequence) sequence (nucleotides 38-518) (**B**) (3D structure was predicted using the RNAcomposer web-tool [87,88] and it was then visualized using the Accelrys Discovery Studio).

The NM001293231 mRNA, is a 1706 nucleotide long transcript. It consists of two exons, where the first ranges from Nucleotides 1 to 194 and the second from Nucleotides 195 to 1706, while the gene coding region is from Nucleotide 38 to 799. Similarly, we have also calculated the secondary structure of the transcript, which is shown in Figure 10. As in the case of NM001293228, there are no known relations of the secondary structure of this transcript to neuroblastoma progression and ontogenesis or to other molecular functions. Since this transcript was smaller, we were able to model the 3D structures of exon 1 (Figure 11A) as well as part of the CDS region of the transcript (Figure 11B). Similarly to the NM001293228 transcript, the prediction of the secondary structure required around 10 h with the same hardware configuration as aforementioned. There are no known properties for the functional role of the 3D MYCN’s RNA structure, which makes it an interesting topic for future research.

#### 2.2.3. MYCN Proto-Oncogene, bHLH Transcription Factor (MYCN), Transcript Variant 2, mRNA

**Figure 12:**
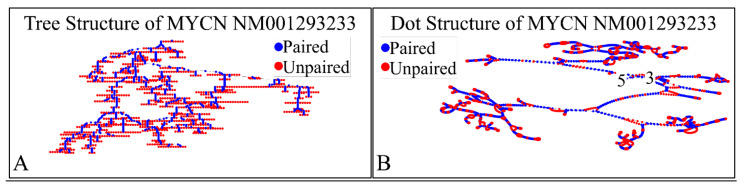
The predicted secondary structure of the *MYCN*, NM001293233 transcript. In particular, the tree structure (**A**) and the dot structure (**B**) of the transcript are presented (predictions were performed with the Matlab^®^ (The Mathworks, Inc. Natick, MA, USA), using the RNAfold method).

**Figure 13:**
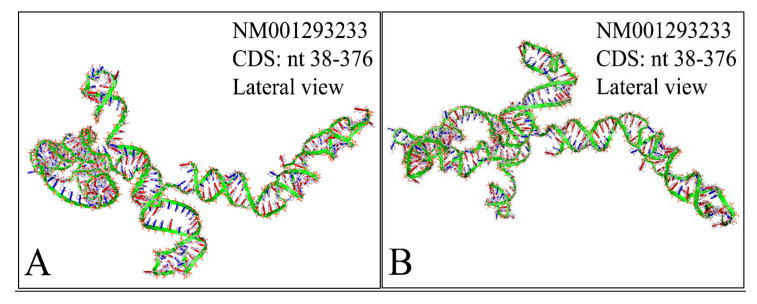
The predicted 3D structure of the *MYCN*, NM001293233 CDS region (nucleotides 38–376). The proximal (**A**) and the distal (**B**) lateral views are presented (3D structure was predicted using the RNAcomposer web-tool [87,88] and it was then visualized using the Accelrys Discovery Studio).

The NM001293233 mRNA, is a 2613 nucleotide long transcript. It consists of three exons, where the first ranges from Nucleotide 1 to 194, the second from Nucleotide 195 to 1101 and the third from Nucleotide 1102 to 2613. The gene coding region is from nucleotide 38 to 376. Similarly, we have also calculated the secondary structure of the transcript, which is shown in Figure 12. As in the case of the previous transcripts, there are no known relations of the secondary structure of this transcript to neuroblastoma progression and ontogenesis or to other molecular functions. In the case of this transcript the CDS region is 339 nucleotides long and we were able to predict the complete 3D structure of it (Figure 13). There are no known properties for the functional role of the 3D MYCN’s RNA structure, which makes it an interesting topic for future research. In addition, this variant lacks the Segment 1b in the 5′ region, compared to Variant 1 [89]. This variant has two ORFs, the MYCNOT, which is translated from the upstream ORF. The MYCNOT has been found to be a long non-coding RNA embedded within the MYCN gene [90]. Further on, this variant (along with the following NM005378 variant) are suspected to participate in the G-Quadraplex structures, formed by the MYCN gene, and thus probably regulating its functions in cell physiology. A recent report has highlighted that this transcript variant probably is responsible for the MYCN mRNA expression levels, as in several cases MYCN amplification, is not correlated to high mRNA levels [91]. However, up-to-date its function is still not well-defined, but there is a hint of its role in anti-apoptosis, which is in agreement to the overall role of MYCN in neuroblastoma.

#### 2.2.4. MYCN Proto-Oncogene, bHLH Transcription Factor (MYCN), Transcript Variant 2, mRNA

The NM005378 mRNA, is a 2613 nucleotide long transcript. It is similar to the NM001293233 transcript and consists of three exons, where the first ranges from Nucleotide 1 to 194, the second from nucleotide 195 to 1101 and the third from Nucleotide 1102 to 2613. The gene coding region is from nucleotide 312 to 1706. As in the case of the NM001293233 transcript, this also lacks segment 1b in the 5′ region. It includes two ORFs, where the one variant is translated from the downstream ORF, encoding the same isoform 1. The function of this transcript is unknown, and it is still under investigation. Overall, the transcription regulation of MYCN is considered a very complex phenomenon, since the MYCN transcripts have a very short half-life, making them difficult to study [92].

### 2.3. The “Anatomy” of MYCN’s Protein

The *MYCN* product (MYCN) is a nuclear phosphoprotein, which can activate transcriptionally many genes, either directly (e.g., ID2) or indirectly [45]. A recent DNA microarray analysis suggested there is a link between DNA methylation and *MYCN* gene expression i.e., there is evidence DNA methylation may directly control MYCN oncoprotein levels [42]. Epigenetic silencing of potential tumor suppressor genes as an alternative mechanism in the absence of genetic mutations has not yet been studied systematically in neuroblastomas and needs further investigation.

**Figure 14:**
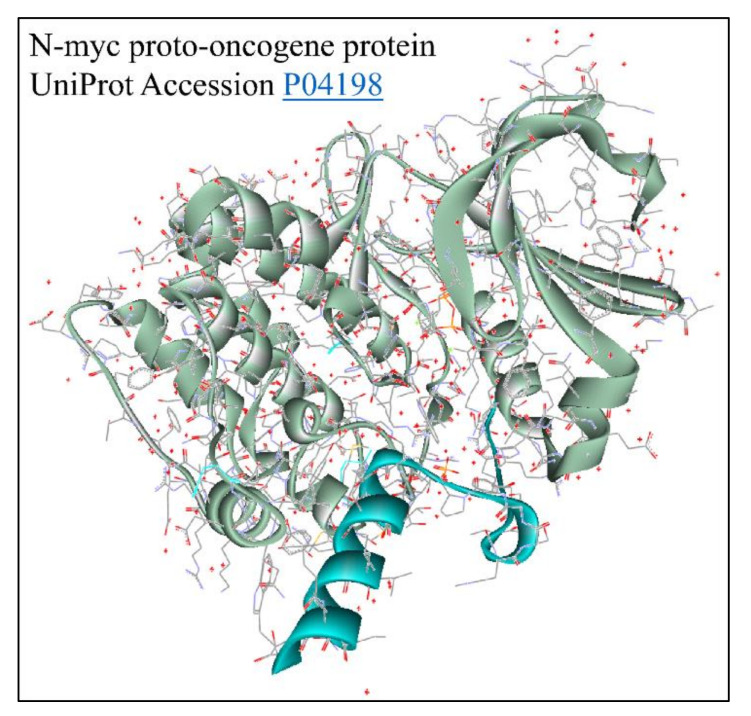
The predicted 3D structure of the MYCN protein with UniProt accession Nr. P04198 [73] (protein was visualized with Accelrys Discovery Studio).

Each of the aforementioned transcripts is registered to provide variants of the MYCN protein. In particular, the NM001293228 produces the NP_001280157.1 N-myc proto-oncogene protein isoform 1, the NM001293231 transcript produces the NP_001280160.1 N-myc proto-oncogene Protein Isoform 2, the NM001293233 transcript produces the NP_001280162.1 N-myc proto-oncogene Protein Isoform 3 and the NM005378 transcript produces the NP_005369.2 N-myc proto-oncogene Protein Isoform 1. There is one reference to the solved structure of the MYCN protein (Figure 14) [73]. This is a member of the MYC family and encodes a protein with a basic helix–loop–helix (bHLH) domain. This protein is located in the nucleus and must dimerize with another bHLH protein in order to bind DNA. Multiple alternatively spliced transcript variants encoding different isoforms have been found for this gene. The NP001280157 variant represents the full-length transcript. “Its Exon 1 includes Segments 1a and 1b (also known as Exon 1a and Exon 1b) encoding Isoform 1” (Provided by https://www.ncbi.nlm.nih.gov/protein/NP_001280157.1, accessed on 5 July 2021) [89]. The NP001280160 variant (transcript variant 3) “lacks Segment 1b and Exon 2, which results in an upstream AUG start codon, as compared to Variant 1. The resulting isoform (Isoform 2) has a shorter and distinct N-terminus, compared to Isoform 1” (provided by https://www.ncbi.nlm.nih.gov/protein/NP_001280160.1, accessed on 5 July 2021). On the other hand, the NP001280162 variant (Transcript Variant 2) lacks Segment 1b in the 5′ region, compared to Variant 1. “This variant includes two open reading frames; the isoform (3, also known as MYCNOT [89]) represented by NM001293233is translated from the upstream open reading frame. The Isoform 3 has an identical N-terminus to that of the Isoform 2, and the function of the Isoform 3 is currently unknown” (provided by https://www.ncbi.nlm.nih.gov/protein/NP_001280162.1, accessed on 5 July 2021). Finally, the NP005369 isoform is derived from the Transcript Variant 2 and lacks Segment 1b in the 5′ region, compared to Variant 1. “This variant includes two open reading frames; Isoform 1 represented by the NM005378 transcript, is translated from the downstream open reading frame. This transcript and Variant 1 encode the same Isoform 1” (provided by https://www.ncbi.nlm.nih.gov/protein/NP_005369.2, accessed on 5 July 2021).

### 2.4. The Cellular Functions of MYCN

One of the first discovered functions of MYCN, was its role in cell cycle regulation. As the cell cycle is a tightly regulated process, MYCN has been found to play a significant role in its regulation. It has been reported that MYCN amplification is linked to the failure of cells to arrest in the G1 phase [93]. This is facilitated by the inhibition of PI3K, which downregulates MYCN protein levels, lowering proliferation and leading to a reduction in S- and M phase cells [94]. Along with PI3K regulation, reduced MYCN expression is correlated to other cell cycle regulators such as the CDK inhibitor p27, E2 factor (E2F) and inhibitor of differentiation 2 (ID2). P27 is known to be upregulated in case of MYCN inhibition, which in turn reduces CDK levels and thus G1-arrest inhibition [95]. Thus, MYCN amplification is directly linked to the aberrant enhancement of cell cycle.

Further on, MYCN has been found to play a “dual role” in the regulation of apoptosis [96]. Recent reports have indicated that MYCN is implicated in the upregulation of the “pro-apoptotic regulator phorbol-12-myristate-13-acetate-induced protein 1” (NOXA). MYCN does not induce apoptosis directly, yet it sensitizes the cell to be more responsive to cytotoxic agents [97]. Therefore, it is known that MYCN inactivation, leads to tumor regression through proliferation arrest and the induction of apoptosis [96]. Interestingly, MYCN is involved in the control of chromatic acetylation levels in the cell, which has been exploited as a therapeutic target since the HDAC inhibitor suberoylanilide hydroxamic acid (SAHA), significantly reduced MYCN levels in *MYCN*-amplified cells, leading to induction of apoptosis [98].

As the regulation of cell cycle and apoptosis, were expected to be the “theatrical stage” for MYCN, metabolism is the prime highlight. As cancer cells require large amounts of energy, they rely to glycolysis for their energy resources, yet another way of surviving comes from glutamine catabolism [99]. Tumors with amplified MYCN have been shown to manifest increased glutamine transport and glutamate metabolism. This has been exploited as a potential therapeutic intervention, where glutamine deprivation in neuroblastoma led to increased cell death [100].

The role of MYCN in cellular physiology is well-studied and there are numerous reports concerning this topic. Yet, it becomes apparent that there is still much to be learned fort its biology.

## 3. Detection of the *MYCN* Gene

Neuroblastoma tumors show remarkable biological heterogeneity. Therefore, to predict the biological behavior of an individual tumor several parameters have been proposed to predict the prognosis of neuroblastoma patients. These include DNA ploidy and deletion of the short arm of Chromosome 1, MYCN gene amplification and TrkA expression and telomerase activity [101]. The use of molecular analysis as a prognostic factor relies on the simplicity, reliability and the rapidity of the chosen procedure. The detection of gene amplification can be carried out using the fluorescence in situ hybridization (FISH) [102] and Southern Blotting (SB) techniques, both of which require a significant amount of high quality DNA and several days in order to obtain the results. Sometimes, the samples obtained by aspiration or biopsy, are small and other times archival paraffin embedded tissue may be used, making it difficult to obtain enough quantity/quality of DNA. In cases where the assay is required in order to determine the appropriate therapeutic regimen the above techniques are unsuitable since they are not rapid.

**Figure 15:**
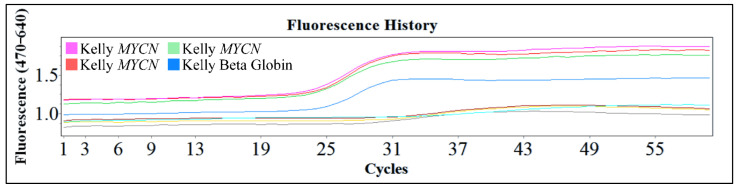
An example of real-time PCR with the Kelly cell line from in-house unpublished data.

One of the popular methods used is the polymerase chain reaction (PCR) and the reverse transcription PCR (RT-PCR). These are powerful procedures for the amplification of small amounts of DNA or mRNA respectively, for molecular analysis. These procedures are efficient since they require only small amounts of sample, they are rapid and in the case of PCR even partially degraded DNA can be used. The downside of these techniques is the fact that in both cases the results are qualitative. Therefore, the exact gene copy number of MYCN cannot be evaluated with the PCR technique. However, the use of this innovative technology, gives the advantage of examining a large number of samples and quantifying the gene and its expression. In Figure 15, we present an example of Real-Time PCR, from unpublished in-house experimentation with the Kelly cell line. This cell line was an ideal model for MYCN amplification, since it is reported to have a ×100 MYCN amplification [2,4,5].

## 4. Molecular Mechanistics of the *MYCN* Gene

Neuroblastoma is presented at diagnosis with extreme heterogeneity. This is probably to the fact that neuroblastoma is inherently a complex and heterogeneous tumor from a biological and genetic perspective. A series of genomic lesions have been discovered in neuroblastoma, each depending on the methodological advancements in the course of time. Initially, the first observations concerned the aberrant number of chromosomes, followed by chromosomal aberrations, such as deletions, translocations, fusions and amplifications. Further on, advances in gene expression came to add more information to the knowledge on neuroblastoma’s biology. This kind of knowledge, initially concerning pure research findings, was added to the diagnostic and prognostic tools for the tumor, as well as to the treatment algorithms of neuroblastoma.

### 4.1. Other Molecular Biomarkers Besides MYCN

Ploidy is characteristic of all tumors. In the case of neuroblastoma, tumors are separated into two main categories; the semi-diploid (45% of tumors) and quasi-triploid tumors (55% of tumors). Ploidy has a prognostic significance in patients younger than two years of age. It has been reported that triploid tumors are usually presented with loss of whole chromosomes, whereas diploid or almost-diploid tumors manifest a major impairment of genome stability leading to chromosomal rearrangements, such as unbalanced permutations. In general, it is reported that triploid tumors have a better prognosis [103,104].

Aside from MYCN aberrations, there are several other chromosomal abnormalities present in neuroblastoma. A much known chromosomal aberration is the 1p deletion, which is often associated with *MYCN* amplification, but it is also frequent without it. It affects the 1p36 region and it is found in the 23–35% of all neuroblastic tumor cases. It is associated with patient’s unfavorable prognosis, where in some cases it remains an independent prognostic indicator, especially in localized, non-metastatic tumors. It is possible that neuroblastoma tumor suppressor oncogenes are located in the 1p36 region and their deletion favors oncogenesis. Such genes include the *CHD5* and *KIF1B* genes, as previously reported [105,106,107,108].

Another chromosomal aberration includes the 11q deletion and in particular deletion of the 11q23 region. This type of chromosomal aberration, is found in the 26–44% of neuroblatoma patients and is inversely related to *MYCN* amplification. This finding divides neuroblastoma patients to two large categories; those with MYCN amplification and those with 11q23, indicating that these two aberrations are mutually exclusive. This aberration, is known to have prognostic value, especially for low- and intermediate-risk neuroblastoma. In this region, the CADM1 gene is located, another tumor suppressor gene, whose deletion is probably linked to neuroblastoma progression [106,107,109,110].

Similarly to the previous deletions, neuroblastoma manifests a deletion in the 3p region, which is known to co-exist with the 11q23 deletion. Both deletions are known, up-to-date, to be present without a *MYCN* amplification or a 1p36 deletion. In addition, this deletion is more frequent in patients of older diagnostic age, indicating that it is probable a distant event in the process of oncogenesis. Similarly to the aforementioned aberrations, this region hosts the RASSF1A gene, whose methylation is also known to be linked to tumor progression [107].

A frequent chromosomal aberration of neuroblastoma is the duplication of the 17q region. It occurs in the 80% of all neuroblastoma cases and is often the result of unbalanced shifts on chromosomes 1p or 11q. Genes located in that region include the *NME1*, *NME2* and *PPM1D* genes (91). In the previous aberrations, it was hypothesized that tumor progression was caused due to the deletions of tumor suppressor genes, while in the present case tumor progression is probably connected to the amplification of oncogenes.

Gene mutations in neuroblastoma have been largely studied and the candidate genes are countless. However, an interesting case is presented with the mutations of the *ALK* gene. These mutations are found to be present in 15% of all sporadic neuroblastoma cases. The *ALKF1174L* mutation has been shown to enhance the oncogenic activity of the amplified *MYCN* oncogene. The *ALK* gene codes for a tyrosine kinase and is found not only in neuroblastoma but also in lung malignancies and lymphomas. Mutations of the *ALK* gene and the signal transduction pathways involving it, probably induce oncogenesis and are thought as possible therapeutic targets [111,112,113,114,115,116,117,118]. In addition to *MYCN* mutations analysis in neuroblastoma, gene expression studies have been used in order to classify the tumor. For example, the expression footprint of six neuroblastoma-related genes (*ALK*, *BIRC5*, *CCND1*, *MYCN*, *NTRK1* and *PHOX2B*) have been used to differentiate neuroblastoma molecular subtypes [119].

### 4.2. Epigenetic Regulation of the MYCN Gene

Besides the aforementioned chromosomal aberrations, much is known on the epigenetic regulation of MYCN in neuroblastoma. Epigenetic regulation of MYCN, includes its interaction with miRNA, as well as the methylation status of the gene. It is known that MYCN is targeted by miRNAs, suppressing MYCN protein expression [107,117,120]. In this section we will deal with the current knowledge on the interactions between MYCN and miRNAs, as well as its epigenetic regulation through methylation.

#### 4.2.1. MYCN and miRNAs

MiRNAs are small single-stranded non-coding RNA molecules (of about 22 nucleotide long) found in all organisms and function as RNA silencing molecules, regulating gene expression post-transcriptionally [121]. MiRNAs bind to their RNA-targets via the nucleotide complementarity principle [122]. The result from the interaction between miRNAs and mRNAs is the silencing of the second, through three main processes; The first concerns mRNA cleavage, the second concerns the destabilization of the mRNA through shortening of its poly(A) tail, and finally the third concerns the inhibition of translation in the ribosomes [123]. Similarly, the MYCN gene is known to be regulated by miRNAs [124]. A search for possible miRNA targets of MYCN revealed a total of 234 miRNAs, using the miRDB database [125,126,127,128], which we present in the Supplementary Materials Table S1. Since the MYCN gene is known to be amplified in neuroblastoma it is expected that most miRNAs, would be negative regulators of the gene. We have searched each miRNA separately for its known roles in neuroblastoma and with respect to MYCN. The details of the predicted miRNAs were searched with the miRBase web-tool [129,130,131,132,133,134].

The majority of miRNAs are reported to be down-regulated in neuroblastoma and in particular, they are found to directly suppress MYCN expression (Table 3). Therefore, in all neuroblastoma cases those miRNAs were found to be down-regulated. Some interesting observations were remarked for several miRNAs, which were found to be up-regulated in neuroblastoma and with a direct link to MYCN. In particular, the miR-17~92 cluster, which includes the miR-17, miR-18a, miR-19a, miR-20a, miR-19b-1 and miR-92a-1 members [135] (Table 3). Those miRNAs, are reported to be up-regulated in neuroblastoma and in particular their over-expression is facilitated by MYCN. It is possible that those miRNAs are enhanced by the gene by an indirect mechanism that attempts to promote tumor progression.

#### 4.2.2. MYCN Methylation and Methylation-Related Mechanisms

Gene methylation is known to play an extremely important role in all parts of cellular and tissue physiology. Similarly, several studies have been conducted for the role of methylation in neuroblastoma and MYCN. Up to date, there are not much data on the methylation status of MYCN in neuroblastoma. Yet, there are several reports concerning the role of methylation of other genes in neuroblastoma, in both tumors with amplified MYCN or not. One of the main findings on neuroblastoma is that the tumor is characterized by the extensive methylation of many tumor suppressor genes [159]. This is the case in almost all neuroblastoma cases irrespectively of MYCN amplification. Interestingly, a recent report has highlighted that miRNAs also have been found methylated in neuroblastoma cell lines, hinting towards their role in tumor progression [160]. Some of these miRNAs included LET7G, MIR124-2, MIR1490, MIR15599, MIR23B, MIR24-1, MIR27B, MIR34C, MIR34B2 and MIR196A-1 [160] (Table 4). In almost all cases gene promoter methylation was mainly related to tumor progression and poor prognosis (Table 4). We have found two exceptions to this rule. The first concerned the NR4A3 gene and actually the Nr4a3 gene in a mouse model (Table 4). A recent study has highlighted that cells with MYCN amplification were found to have a hypo-methylation pattern of the NR4A3 gene’s Exon 3, while those tumors with a methylated Exon 3, manifested potentially better prognosis [161]. In addition, three different studies indicated another exception, concerning the CD44 gene (Table 4). In both MYCN amplified and non-amplified neuroblastoma, CD44 was found to play a dual role that is when un-methylated was manifesting high expression levels, redirecting to tumor suppression, while when hyper-methylated it was related to tumor progression [162,163,164].

#### 4.2.3. Other Epigenetic and Post-Translational Mechanisms in Neuroblastoma

Up to date there are not many studies concerning the role of other epigenetic (e.g., acetylation) or post-translational mechanisms in neuroblastoma. The existent studies have investigated this phenomenon through the application of inhibitors and by observing the outcome. For example, a recent study has found that the inhibitor SF1126, which inhibits BRD4 bromodomain binding to acetylated lysine residues with histone H3 as well as PI3K activity in a MYCN amplified neuroblastoma cell line, manifested tumor inhibitory effects and was proposed as a potential therapeutic agent [194].

In another interesting study, it has been proposed that a known anti-depressant, fluoxetine, was able to induce apoptosis in neuroblastoma cells. Interestingly, fluoxetine was able to induce apoptosis through two mechanistic pathways; the caspase cascade and the probably the hyper-acetylation of histone H3 and H4, upregulation of p300 histone acetyltransferase (HAT), as well as the downregulation of histone deacetylases (HDAC) [195]. However, this was observed in MYCN amplified cells, while it was not apparent in MYCN non-amplified or MYCN knock-down cells [195]. The results from this study, indicated that MYCN is actively participating in other post-transcriptional and post-translational modifications. The case of fluoxetine, is of great interest since it is a substance that is used for the treatment of different conditions. In the same context, another recent work indicated the use of anti-diabetic drugs, such as metformin and phenformin, in the treatment of neuroblastoma. The treatment of neuroblastoma cells, both with or without MYCN amplification, with metformin and phenformin resulted to increased apoptosis rates and inhibition of cell proliferation. One the possible mechanisms through which these drugs act is the augmentation of H3 acetylation [196].

Another study, indicated that the drug romidepsin, a selective histone deacetylase inhibitor (HDAC1/2), was able to induce apoptosis to neuroblastoma cells. In this study, it appeared that neuroblastoma progression is probably linked to the extensive histone acetylation and its inhibition was able to destabilize tumor progression [197]. Further on, it has been reported that MYCN and HDAC genes are functioning in cooperation in neuroblastoma. At the same time another gene the Grainyhead-like 1 (GRHL1), which is known to be critical in Drosophila neural development, was found to be one of the main targets of HDAC inhibitor treatment in neuroblastoma [198]. It was found that an increase in the histone H4 “pan-acetylation” in association with its promoter was followed by extensive transcriptional activation [198]. The HDAC3 and MYCN genes are physically co-localized to the GRHL1 promoter and they are probably able to repress its transcription. In cases of high GRHL1 expression, in neuroblastoma, tumor progression was suppressed, as well as patient survival was more favorable. Thus, it appeared that neuroblastoma with or without MYCN amplification, manifested low proliferation rates and sensitivity to therapy if GRHL1 was present [198].

### 4.3. MYCN Gene Amplification: “The Oldest Trick in the Book”

*MYCN* is known to regulate the proliferation, growth, differentiation and survival of cells of the developing nervous system [199]. Initial karyotyping techniques, has shown that large chromosomal abnormalities are found in the 1p region of Chromosome 1. This finding became known in the mid-1980s, where it was found that this chromosomal aberration was linked to ectopic *MYCN* amplification, which is located on the short arm of chromosome 2p24 [200]. It became apparent that the presence of *MYCN* amplification affected patients with advanced neuroblastoma and manifested an unfavorable prognosis. Numerous studies have shown that *MYCN* overexpression leads to the development of neuroblastic tumors and it seems that this is achieved by regulating p53 levels, regulating key mechanisms of cell apoptosis [201]. In general, although MYCN controls a large number of genes, only a small number of regulatory genes have been studied. Interestingly, the high expression of MYCN-controlled genes is not only characteristic of *MYCN*-amplified tumors but is also found in other high-risk neuroblastic tumors that do not show *MYCN* amplification. However, in addition to *MYCN*, several other cytogenetic abnormalities have been frequently observed in neuroblastic tumors. Most involve the loss of genomic material.

*MYCN* amplification, drives one out of six cases of neuroblastoma. The mechanisms through which amplification takes place are still obscure, yet a recent report indicated that gene amplification is related to a sort of “enhancer high jacking” of the *MYCN* gene [202]. In particular, they have reported that there are two mechanistic approaches for the gene’s amplification. The first includes the co-amplification of the proximal enhancer driven by the noradrenergic core regulatory circuit (CRC), while the second involves the ectopic “enhancer hijacking”, contributing to the loss of local gene regulatory elements [202].

A number of genetic characteristics of neuroblastic tumors have significant prognostic value. These include tumor ploidy, *MYCN* amplification, and for tumors without *MYCN* amplification the type of chromosomal lesions that emerge. Tumors with poly-ploidy have a better prognosis as compared to tumors that are diploid. This is especially true for patients without *MYCN* amplification and of younger age (less than 18 months of age) [203]. On the other hand, triploid tumors are known to have the best prognosis, while tetraploid tumors have a prognosis similar to diploid tumors (inferior) [104]. The most important and most known aberration of neuroblastoma, with an unfavorable prognosis, is *MYCN* amplification.

One of the first discoveries concerning the molecular mechanistic of neuroblastoma, concerned the discovery of *MYCN* amplification. Thirty years ago it was realized that an increase in the *MYCN* copy-number is associated with rapid tumor progression in neuroblastoma [31]. Following that discovery, *MYCN* amplification emerged and it was discovered its relation to the unfavorable outcome of neuroblastoma [203,204,205]. In the meantime, *MYCN* amplification became a “standard operating procedure” in terms of neuroblastoma diagnostics, as well as its categorization [206]. *MYCN* amplification can be detected at any stage of the tumor’s progression, although it is particularly rare in Stage 1 tumors.

The frequency of *MYCN* amplification has been found to be at 25% of all neuroblastomas and in 40% of high-risk neuroblastomas [96,201]. The definition for *MYCN* amplification requires the detection of at least four copies more as compared to the normal alleles. Frequently, MYCN amplification is found in neuroblastoma cell lines, which ranges from 20 to 40 times [200]. From a mechanistic point of view, it has been reported that MYCN activity is probably related to *NM23-H1* and *NM23-H2* genes located in the 17q region [207]. In addition, *MYCN* expression has been reported to be linked to activation by the *TP53* gene and thus regulating the escape from apoptosis of neuroblastic cells [201]. Another important molecular event thought to participate in *MYCN* amplification is the presence of *ALK* mutations in neuroblastoma [112]. Interestingly, along with MYCN amplification, ALK has also been reported to be amplified in neuroblastoma [208,209]. In addition, it has been found that in tumors without *MYCN* amplification a number of other chromosomal aberrations are present. Such examples include the gain or deletion of complete chromosomes such as gain of Chromosome 17, Chromosome 7, Chromosome 2 and deletions of Chromosomes 3, 4, and 14 [210]. Similarly, it is possible to observe the gain or deletion of chromosomal regions, such as the loss of Location 1p of Chromosome 1, 11q on Chromosome 11, 14q on Chromosome 14 and 3p in Chromosome 3. Similarly, most frequent chromosomal gains are the 17q and 2p [210]. The latter, is also known to be present with ALK amplification. The deletion of specific chromosomal regions is known to be present in neuroblastoma progression due to the concurrent deletion of tumor suppressor genes that is these specific regions are the loci of tumor suppressors and their absence favors tumor progression. Such examples are the 1p, 3p and 11q regions. Similarly, the gain of chromosomal regions is tightly related to the amplification of oncogenes, favoring tumor ontogenesis [211,212,213]. An older report has indicated that the presence of complete chromosomal loss or gain was related to more favorable outcome, as compared to the loss or gain of specific chromosomal regions [214,215].

### 4.4. Therapeutic Interventions with MYCN

One of the first interventions in the treatment of neuroblastoma is chemotherapy, but of equal value comes surgical intervention and removal. Surgery is not always the first choice, as neuroblastoma manifests a diffuse character during its growth, making surgery impossible. Yet, there are several factors that can modify the decision for surgical intervention. All recent reports agree on the fact that disease staging is crucial for the treatment of the disease [47,216]. It has been reported that surgical treatment and its success is linked to the image defined risk factors (IDRF), which include the crossing and extending of the tumor, the engulfment of vessels, the compression of other organs and the encasing of nerves [217]. It would be interesting to find molecular factors that could lead to the decision for surgical intervention, yet to the best of our knowledge, none exist up-to-date.

Since the discovery of MYCN amplification in neuroblastoma, it became apparent that MYCN could pose an attractive therapeutic target. The first report including MYCN as potential target came by Lu et al. (2003) [218]. In the time course of research, it became evident that simultaneous inhibition of multiple targets could prove useful in the treatment of neuroblastoma. For example, a recent study has reported that the simultaneous inhibition of MYCN and mTOR using bromodomain extra-terminal protein inhibitors and temsirolimus manifested significant cell survival suppression [219]. Another study showed that the use of PARP and CHK1 inhibitors synergized to induce death in neuroblastoma cells and in primary cultures of MYCN amplified and MYCN overexpressing cells [220]. In agreement with our previous comments for the role of MYCN in cellular metabolism, a recent study highlighted this approach as a possible therapeutic intervention. In particular, they have reported that the pyrimidine biosynthetic enzyme dihydroorotate dehydrogenase (DHODH) consisted of a potent therapeutic target for MYCN-amplified neuroblastoma. Thus, they have shown that inhibition of DHODH suppressed the proliferation of MYCN-amplified neuroblastoma cells [221]. Up-to-date there are numerous studies concerning the use of inhibitors in neuroblastoma, either directly on MYCN, as well as indirectly through the inhibition of other molecules. We have summarized the most recent inhibitors and molecules in Table 5.

## 5. Conclusions

It the present study, we have reviewed the literature for the connection between MYCN and neuroblastoma. We have investigated mostly all possible aspects in MYCN biology including, the presence of MYCN G-quadraplexes in the gene’s structure, the “anatomy” of MYCN’s transcripts, as well as the epigenetic regulation in relation to neuroblastoma. MYCN, is one the most well studied genes in neuroblastoma and yet, there is much to be learned on its biology. We have seen that the ontogeny of neuroblastoma is closely related to MYCN biology, where the presence of chromosomal aberrations is tightly linked to the deletion or the gain of tumor suppressor and oncogenes respectively. MYCN, is known to play a significant role in the chromosomal stability of neuroblastoma, as well as it is able to change the methylation and acetylation status of the genome and the genome-regulatory mechanisms. In that sense, several approaches have attempted to exploit the properties of MYCN and its biology, in order to use them as prognostic and more importantly therapeutic targets. It is certain that we still need much more research in order to comprehend the depth of the disease and exploit that knowledge for its therapy.

## Figures and Tables

**Table 1:** The analysis of the *MYCN* gene (table has been reproduced from the NCBI provided data https://www.ncbi.nlm.nih.gov/gene/4613, accessed on 20 June 2021.).

Type	Start	Stop	Strand	Comment
source	1	6455	5′→3′	/gene = MYCN /gene_synonym = MYCN-AS1; N-CYM; NCYM; NYCM /organism = Homo sapiens /mol_type = genomic DNA /db_xref = taxon:9606 /chromosome = 2
gene	<1C	1884	3′→5′	/note = MYCN opposite strand; Derived by automated computational analysis /db_xref = GeneID:10408 /db_xref = HGNC:HGNC:16911 /db_xref = MIM:605374
ncRNA	<425C 983C	548 1136	3′→5′	/ncRNA_class = lncRNA /product = MYCN opposite strand, transcript variant 1 /transcript_id = NR_110230.2
ncRNA	<425C 983C	548 1884	3′→5′	/ncRNA_class = lncRNA /gene = MYCNOS /gene_synonym = MYCN-AS1; N-CYM; NCYM; NYCM /product = MYCN opposite strand, transcript variant 3 /transcript_id = NR_161163.1
ncRNA	425C 983C	548 1136	3′→5′	/ncRNA_class = lncRNA /gene = MYCNOS /gene_synonym = MYCN-AS1; N-CYM; NCYM; NYCM /product = MYCN opposite strand, transcript variant 2 /transcript_id = NR_161162.1
mRNA	1 1399 4944	504 2305 6455	5′→3′	/gene_synonym = bHLHe37; MODED; N-myc; NMYC; ODED /product = MYCN proto-oncogene, bHLH transcription factor, transcript variant 1 /transcript_id = NM_001293228.2
mRNA	1 1399 4944	194 2305 6455	5′→3′	/gene = MYCN /gene_synonym = bHLHe37; MODED; N-myc; NMYC; ODED /product = MYCN proto-oncogene, bHLH transcription factor, transcript variant 2 /transcript_id = NM_005378.6 /db_xref = Ensembl:ENST00000281043.4
mRNA	1 1399 4944	194 2305 6455	5′→3′	/gene = MYCN /gene_synonym = bHLHe37; MODED; N-myc; NMYC; ODED /product = MYCN proto-oncogene, bHLH transcription factor, transcript variant 2 /transcript_id = NM_001293233.2
mRNA	1 4944	194 6455		/gene = MYCN /gene_synonym = bHLHe37; MODED; N-myc; NMYC; ODED /product = MYCN proto-oncogene, bHLH transcription factor, transcript variant 3 /transcript_id = NM_001293231.2
CDS	38 4944	194 5548	5′→3′	/gene = MYCN /gene_synonym = bHLHe37; MODED; N-myc; NMYC; ODED /note = isoform 2 is encoded by transcript variant 3; /codon_start = 1 /product = N-myc proto-oncogene protein isoform 2 /protein_id = NP_001280160.1 /db_xref = CCDS:CCDS86823.1 /translation=MRGAPGNCVGAEQALARRKRAQTVAIRGHPRPPGPPGDTRAESPPDPLQSAGDDEDDEEEDEEEEIDVVTVEKRRSSSNTKAVTTFTITVRPKNAALGPGRAQSSELILKRCLPIHQQHNYAAPSPYVESEDAPPQKKIKSEASPRPLKSVIPPKAKSLSPRNSDSEDSERRRNHNILERQRRNDLRSSFLTLRDHVPELVKNEKAAKVVILKKATEYVHSLQAEEHQLLLEKEKLQARQQQLLKKIEHARTC
CDS	38 1399	194 1580	5′→3′	/gene = MYCN /gene_synonym = bHLHe37; MODED; N-myc; NMYC; ODED /note = isoform 3 is encoded by transcript variant 2; /codon_start = 1 /product = N-myc proto-oncogene protein isoform 3 /protein_id = NP_001280162.1 /db_xref = GeneID:4613 /db_xref = HGNC:HGNC:7559 /db_xref = MIM:164840 /translation=MRGAPGNCVGAEQALARRKRAQTVAIRGHPRPPGPPGDTRAESPPDPLQSAGVLEVGAGPRLPRPPREGSTPGIKTNGAERSPQSPAGRRADAELLHVHHAGHDLQEPRPRV
CDS	1516 4944	2305 5548	5′→3′	/gene = MYCN /gene_synonym = bHLHe37; MODED; N-myc; NMYC; ODED /note = isoform 1 is encoded by transcript variant 2; /codon_start = 1 /product = N-myc proto-oncogene protein isoform 1 /protein_id = NP_005369.2 /db_xref = CCDS:CCDS1687.1 /db_xref = Ensembl:ENSP00000281043.3 /db_xref = GeneID:4613 /db_xref = HGNC:HGNC:7559 /db_xref = MIM:164840 /translation=MPSCSTSTMPGMICKNPDLEFDSLQPCFYPDEDDFYFGGPDSTPPGEDIWKKFELLPTPPLSPSRGFAEHSSEPPSWVTEMLLENELWGSPAEEDAFGLGGLGGLTPNPVILQDCMWSGFSAREKLERAVSEKLQHGRGPPTAGSTAQSPGAGAASPAGRGHGGAAGAGRAGAALPAELAHPAAECVDPAVVFPFPVNKREPAPVPAAPASAPAAGPAVASGAGIAAPAGAPGVAPPRPGGRQTSGGDHKALSTSGEDTLSDSDDEDDEEEDEEEEIDVVTVEKRRSSSNTKAVTTFTITVRPKNAALGPGRAQSSELILKRCLPIHQQHNYAAPSPYVESEDAPPQKKIKSEASPRPLKSVIPPKAKSLSPRNSDSEDSERRRNHNILERQRRNDLRSSFLTLRDHVPELVKNEKAAKVVILKKATEYVHSLQAEEHQLLLEKEKLQARQQQLLKKIEHARTC
misc_feature	1570	1656	5′→3′	/gene = MYCN /gene_synonym = bHLHe37; MODED; N-myc; NMYC; ODED /note = propagated from UniProtKB/Swiss-Prot (P04198.2); Region: Interactio nwith AURKA./evidence=ECO:0000269|PubMed:27837025
misc_feature	1696	1782	5′→3′	/gene = MYCN /gene_synonym = bHLHe37; MODED; N-myc; NMYC; ODED /note = propagated from UniProtKB/Swiss-Prot (P04198.2); Region: Interaction with AURKA and FBXW7./evidence=ECO:0000269|PubMed:27837025
misc_feature	2296	2298	5′→3′	/gene = MYCN /gene_synonym = bHLHe37; MODED; N-myc; NMYC; ODED /note = Phosphoserine, by CK2./evidence=ECO:0000269|PubMed:1425701; propagated from UniProtKB/Swiss-Prot (P04198.2); phosphorylation site
misc_feature	2302	2304	5′→3′	/gene = MYCN /gene_synonym = bHLHe37; MODED; N-myc; NMYC; ODED /note = Phosphoserine, by CK2./evidence=ECO:0000269|PubMed:1425701; propagated from UniProtKB/Swiss-Prot (P04198.2); phosphorylation site
misc_feature	5450	5515	5′→3′	/gene = MYCN /gene_synonym = bHLHe37; MODED; N-myc; NMYC; ODED /note = propagated from UniProtKB/Swiss-Prot (P04198.2); Region: Leucine-zipper
CDS	1516 4944	2305 5548	5′→3′	/gene = MYCN/ gene_synonym = bHLHe37; MODED; N-myc; NMYC; ODED /note = isoform 1 is encoded by transcript variant 1; /codon_start = 1 /product = N-myc proto-oncogene protein isoform 1/protein_id = NP_001280157.1 /db_xref = CCDS:CCDS1687.1 /translation=MPSCSTSTMPGMICKNPDLEFDSLQPCFYPDEDDFYFGGPDSTPPGEDIWKKFELLPTPPLSPSRGFAEHSSEPPSWVTEMLLENELWGSPAEEDAFGLGGLGGLTPNPVILQDCMWSGFSAREKLERAVSEKLQHGRGPPTAGSTAQSPGAGAASPAGRGHGGAAGAGRAGAALPAELAHPAAECVDPAVVFPFPVNKREPAPVPAAPASAPAAGPAVASGAGIAAPAGAPGVAPPRPGGRQTSGGDHKALSTSGEDTLSDSDDEDDEEEDEEEEIDVVTVEKRRSSSNTKAVTTFTITVRPKNAALGPGRAQSSELILKRCLPIHQQHNYAAPSPYVESEDAPPQKKIKSEASPRPLKSVIPPKAKSLSPRNSDSEDSERRRNHNILERQRRNDLRSSFLTLRDHVPELVKNEKAAKVVILKKATEYVHSLQAEEHQLLLEKEKLQARQQQLLKKIEHARTC
misc_feature	1570	1656	5′→3′	/gene = MYCN /gene_synonym = bHLHe37; MODED; N-myc; NMYC; ODED /note = propagated from UniProtKB/Swiss-Prot (P04198.2); Region: Interaction with AURKA./evidence=ECO:0000269|PubMed:27837025
misc_feature	1696	1782	5′→3′	/gene = MYCN /gene_synonym = bHLHe37; MODED; N-myc; NMYC; ODED /note = propagated from UniProtKB/Swiss-Prot (P04198.2); Region: Interaction with AURKA and FBXW7./evidence=ECO:0000269|PubMed:27837025
misc_feature	2296	2298	5′→3′	/gene = MYCN /gene_synonym = bHLHe37; MODED; N-myc; NMYC; ODED /note = Phosphoserine, by CK2./evidence=ECO:0000269|PubMed:1425701; propagated from UniProtKB/Swiss-Prot (P04198.2); phosphorylation site
misc_feature	2302	2304	5′→3′	/gene = MYCN /gene_synonym = bHLHe37; MODED; N-myc; NMYC; ODED /note = Phosphoserine, by CK2./evidence=ECO:0000269|PubMed:1425701; propagated from UniProtKB/Swiss-Prot (P04198.2); phosphorylation site
misc_feature	5450	5515	5′→3′	/gene = MYCN /gene_synonym = bHLHe37; MODED; N-myc; NMYC; ODED /note = propagated from UniProtKB/Swiss-Prot (P04198.2); Region: Leucine-zipper

**Table 2:** TATA-Boxes on the *MYCN* gene.

Inv.	Location (5′→3′)	Subsequence	Pattern
1	3930	TATA-box	TATAAAA
2	4401	TATA-box	ATATATA
3	4402	TATA-box	TATATAT
4	4403	TATA-box	ATATATA
5	4882	TATA-box	ATATATA
6	4883	TATA-box	TATATAT
7	6271	TATA-box	TATATAT
8	6272	TATA-box	ATATATA
9	6273	TATA-box	TATATAT
10	6274	TATA-box	ATATATA
11	6275	TATA-box	TATATAT
12	6382	TATA-box	TATATAT

**Table 3:** MYCN-related miRNAs. Out of 234 miRNAs targeting MYCN, twenty were studied for their role in neuroblastoma.

miRNA	Symbol	Accession	Relation between miRNA and MYCN	miRNA Expression in Neuroblastoma	Effect on Therapy-Related Resistance	Citation
hsa-miR-101-3p	MIR101-1	MI0000103	MIR101-1 directly suppresses MYCN	Down-regulated	Unknown	Buechner et al. (2011) [136]
hsa-miR-449a	MIR449A	MI0001648	MIR449A directly suppresses MYCN	Down-regulated	Unknown	Zhao et al. (2016) [137]
hsa-miR-34a	MIR34A	MI0000268	MIR34A directly suppresses MYCN	Down-regulated	Induces chemo- and radiosensitivity	Cole et al. (2008) [138] Di Paolo et al. (2020) [139] Soni et al. (2013) [140] Stallings et al. (2009) [141] Wei et al. (2008) [142]
hsa-miR-202	MIR202	MI0003130	MIR202 directly suppresses MYCN	Down-regulated	Unknown	Li et al. (2014) [143]
hsa-miR-335-3p	MIR335	MI0000816	MIR335 directly suppresses MYCN	Down-regulated	Unknown	Lynch et al. (2012) [144]
hsa-miR-144-3p	MIR144	MI0000460	MIR144 directly suppresses MYCN	Down-regulated	Induces chemo- and radiosensitivity	Liu et al. (2019) [145]
hsa-miR-107	MIR107	MI0000114	MIR107 directly suppresses MYCN	Down-regulated	Induces apoptosis, cell cycle arrest, chemosensitivity	Ramaiah et al. (2013) [146]
hsa-miR-29c	MIR29C	MI0000735	MIR29C indirectly suppresses MYCN	Down-regulated	Unknown	Gan et al. (2016) [147]
hsa-miR-7	MIR7	MI0000127	MIR7 probably enhances MYCN indirectly	Up-regulated	Unknown	Cheung et al. (2014) [148]
hsa-miR-29a-3p	MIR29A	MI0000087	MIR29A directly suppresses MYCN	Down-regulated	Unknown	Cheung et al. (2014) [148]
hsa-miR-29b-3p	MIR29B	MI0000105	MIR29B directly suppresses MYCN	Down-regulated	Unknown	Teitz et al. (2013) [149]
hsa-miR-98-5p	MIR98	MI0000100	MIR98 directly suppresses MYCN	Down-regulated	Unknown	Cheng et al. (2020) [150]
hsa-miR-145-5p	MIR145	MI0000461	MIR145 directly suppresses MYCN	Down-regulated	Unknown	Zhao et al. (2020) [151]
hsa-miR-19a-3p	MIR19A	MI0000073	MYCN enhances MIR19A	Up-regulated	Represses neuronal differentiation	Loven et al. (2010) [152]
hsa-miR-19b-3p	MIR19B	MI0000074	MIR19B directly suppresses MYCN	Down-regulated	Unknown	Kumps et al. (2013) [72] De Brouwer et al. (2012) [71]
hsa-miR-193b-3p	MIR193B	MI0003137	MIR193B directly suppresses MYCN	Down-regulated	Induces apoptosis, proliferation arrest	Roth et al. (2018) [153]
hsa-miR-200b-3p	MIR200B	MI0000342	MIR200B directly suppresses MYCN	Down-regulated	Induces apoptosis, cell cycle arrest	Ramaiah et al. (2013) [146]
hsa-miR-106a-5p	MIR106A	MI0000113	MYCN enhances MIR106A	Up-regulated	Promotes cell proliferation	Schulte et al. (2008) [154]
hsa-miR-20a-5p	MIR20A	MI0000076	MYCN enhances MIR20A	Up-regulated	Promotes cell proliferation	Samaraweera et al. (2017) [155]
hsa-miR-17-5p	MIR17	MI0000071	MYCN enhances MIR17	Up-regulated	Promotes cell proliferation	Claeys et al. (2019) [156] Samaraweera et al. (2017) [155] Naraparaju et al. (2016) [157] Buechner et al. (2011) [158] Loven et al. (2010) [152] Schulte et al. (2008) [154]

**Table 4:** *MYCN*-related methylation in neuroblastoma.

Gene	Methylation Status	MYCN Amplification	Effect on Neuroblastoma	Effect on Prognosis	Effect on Therapy-Related Resistance	Citation
FOXR2	Methylated	Non-amplified	Tumor progression	Poor	Unknown	Korschunov et al. (2021) [165] Schmitt-Hoffner et al. (2021) [166]
VRK1	Hypo-methylated	Amplified	Tumor progression	Poor	Unknown	Colmenero-Repiso et al. (2020) [167]
DDX58	Hyper-methylated	Both	Tumor progression	Poor	Chemo-resistance	Lin et al. (2020) [168]
H3K9me1/2	Methylated	Both	Tumor progression	Poor	Chemo-resistance. De-methylation confers tumor sensitivity	Gupta et al. (2018) [169]
NAV2, NCAM2, PRPH	Hyper-methylated	Amplified	Tumor progression	Poor	Chemo-resistance. De-methylation confers tumor sensitivity	Westerlund et al. (2017) [159]
H3K79	Methylated	Amplified	Tumor progression	Poor	Chemo-resistance. De-methylation confers tumor sensitivity	Wong et al. (2017) [170]
LET7G, MIR124-2, MIR1490, MIR15599, MIR23B, MIR24-1, MIR27B, MIR34C, MIR34B2, MIR196A-1	Methylated	Both	Tumor progression	Poor	Chemo-resistance. De-methylation confers tumor sensitivity	Parodi et al. (2016) [160]
NCYM	Methylated	Both	Tumor progression	Poor	Unknown	Liu et al. (2016) [171]
PCDHB, ABCB1, CACNA1G, CD44,DUSP23, PRDM2, RBP1, SFRP1	Hyper-methylated	Both	Tumor progression	Poor	Chemo-resistance. De-methylation confers tumor sensitivity	Henrich et al. (2016) [172]
KRT19, PRPH, CNR1, QPCT	Methylated	Both	Tumor progression	Poor	Chemo-resistance. De-methylation confers tumor sensitivity	Henrich et al. (2016) [172]
CD9	Methylated	Non-amplified	Tumor progression	Poor	Unknown	Fabian et al. (2016) [173]
TFAP2B	Methylated	Both	Tumor progression	Poor	Chemo-resistance. De-methylation confers tumor sensitivity	Ikram et al. (2016) [174]
H3K4	Tri-methylated	Both	Tumor progression	Poor	Unknown	Sun et al. (2015) [175]
NNAT, TP73, CCND, RUNX32, CTSZ, DUSP2, HPN, JAK2, LRRC4, MAGEA2, MGMT, PAX8, ECRG4, RB1, TDGF1, TSPAN32	Hyper-methylated	Both	Tumor progression	Poor	Unknown	Yanez et al. (2015) [176] Gonzalez-Gomez et al. (2003) [177]
p19-INK4d	Hyper-methylated	Both	Tumor progression	Poor	Unknown	Dreidax et al. (2014) [178]
RASSF1A, PCDHB	Methylated	Both	Tumor progression	Poor	Unknown	Haruta et al. (2014) [179] Lazcoz et al. (2007) [180] Yang et al. (2004) [181]
NR4A3	Hyper-methylated Hypo-methylated	Both	Tumor inhibition Tumor Progression	Good Poor	Unknown	Uekusa et al. (2014) [161]
CASP8	Methylated	Both	Tumor Progression	Poor	Unknown	Asada et al. (2013) [162] Lazcoz et al. (2007) [180] Fulda et al. (2006) [182] Casciano et al. (2004) [183] Gonzalez-Gomez et al. (2003) [177]
ZAR1	Hyper-methylated	Both	Tumor Progression	Poor	Unknown	Sugito et al. (2013) [184]
CASR	Hyper-methylated	Both	Tumor Progression	Poor	Unknown	Casala et al. (2013) [185]
KRT19, FAS, PRPH, CNR1, QPCT, HIST1H3C, ACSS3, GRB10	Methylated	Both	Tumor Progression	Poor	Unknown	Decock et al. (2012) [186]
HIST1H3C, GNAS	Methylated	Both	Tumor inhibition	Good	Unknown	Decock et al. (2012) [186]
DNAJC15, NTRK1, TNFRSF10D	Methylated	Both	Tumor Progression	Poor	Unknown (found in older patients)	Lau et al. (2012) [187]
DNAJC15, NTRK1, PYCARD	Hyper-methylated	Amplified	Tumor Progression	Poor	Unknown	Lau et al. (2012) [187]
FOLH1, MYOD1, THBS1	Hyper-methylated	Both	Tumor Progression	Poor	Unknown	Lau et al. (2012) [187] Gonzalez-Gomez et al. (2003) [177]
SLC16A5, ZNF206	Hypo-methylated	Both	Unknown	Unknown	Unknown	Sugito et al. (2013) [188]
RASSF family	Methylated	Both	Tumor Progression	Poor	Unknown	Djos et al. (2012) [189] Misawa et al. (2009) [190] Michalowski et al. (2008) [20] Lazcoz et al. (2007) [180] Yang et al. (2004) [181]
GSTP1	Hyper-methylated	Both	Tumor Progression	Poor	Unknown	Gumy-Pause et al. (2012) [191]
CD44, RASSF1A, CASP8, PTEN, ZMYND10, CDH1, PRDM2	Methylated	Both	Tumor Progression	Poor	Unknown	Hoebeeck et al. (2009) [163]
SEMA3B	Methylated	Both	Tumor Progression	Poor	Unknown	Nair et al. (2007) [192]
PTEN, MGMT, MXI1, FGFR2	Methylated	Both	Tumor Progression	Poor	Unknown	Lazcoz et al. (2007) [193]
CD44 expressing CD44 not expressing	Unmethylated Hyper-methylated	Both Both	Tumor inhibition Tumor Progression	Good Poor	Unknown	Yan et al. (2003) [164]

**Table 5:** Therapeutic targets in neuroblastoma.

Target Molecule	Inhibitor	MYCN Amplification	Citation
MYCN Proto-Oncogene, BHLH Transcription Factor (MYCN)	JQ1 OTX-015	Yes	[219]
Mammalian Target of Rapamycin (mTOR)	Temsirolimus Rapamycin	Yes	[219,222,223]
Peroxisome Proliferator-Activated Receptors (PPAR)	Olaparib Talazoparib	Yes	[220]
Checkpoint Kinase 1 (CHK1)	MK-8776 PF-00477736	Yes	[220]
Dihydroorotate Dehydrogenase (DHODH)	Leflunomide Brequinar sodium GSK983	Yes and No	[221]
Tropomyosin Receptor Kinase C (TrkC)	Valproic Acid	Yes	[224]
Neuronal Leucine-Rich Repeat 1 (NLRR1)	Monoclonal Antibodies Against the Extracellular Domains Of NLRR1 (N1mAb)	Yes	[225]
Mitogen-Activated Protein Kinase Kinase Kinase 3 (MAP3K3) Bromodomain-Containing Protein 4 (BRD4)	miR-3140-3p	Yes	[226]
Ornithine Decarboxylase (ODC)	Probenecid DFMO	Yes	[227]
B-Cell Lymphoma 2 (BCL2)	Venetoclax	Yes	[228]
Cyclin Dependent Kinase 4 (CDK4) Cyclin Dependent Kinase 6 (CDK6)	Flavopiridol Roscovitine Dinaciclib P276-00 AT7519 TG02 RoniciclibR GB-286638	Yes	[222,229]
ALK Receptor Tyrosine Kinase (ALK)	Crizotinib Ceritinib Alectinib Brigatinib Entrectinib Lorlatinib		[230]
Androgen Receptor (AR)	Statins Abiraterone Acetate		[231]
Peptidyl-Prolyl Cis/Trans Isomerase (Pin1)	Sulfopin		[232]
Bromodomain-Containing Protein 4 (BRD4)	NHWD-870 BMS-986158 OTX-015 GSK-525762		[233]
Monocarboxylate Transporter 1 (MCT1)	AZD3965	Yes	[234]
Histone Deacetylase (HDAC)	Suberanoyl Hydroxamic Acid (SAHA)	Yes	[235]
Ubiquitin Carboxyl-Terminal Hydrolase 5 (USP5)	SE486-11	Yes	[235]

## Data Availability

Not applicable.

## Supplementary Materials

The following are available online at https://www.mdpi.com/article/10.3390/diseases9040078/s1, Table S1: predicted miRNAs as potential targets for the *MYCN* gene. miRNAs were predicted using the miRDB database [125,126,127,128].
