# Supplementary material for: MYCN in Neuroblastoma: “Old Wine into New Wineskins”
Source: Diseases. 2021 Oct 29;9(4):78. doi: 10.3390/diseases9040078 (PMC8628738; doi:10.3390/diseases9040078)
Supplement: Supplementary file 1 [file diseases-09-00078-s001.zip › diseases-1421446-supplementary.pdf]

Target Rank	Target Score	miRNA Name	Studied for its role in Neuroblastoma and *MYCN*?
1	100	hsa-miR-101-3p	YES
2	100	hsa-miR-5011-5p	NO
3	99	hsa-miR-767-5p	NO
4	98	hsa-miR-449a	YES
5	98	hsa-miR-34a-5p	YES
6	98	hsa-miR-34c-5p	NO
7	98	hsa-miR-4482-5p	NO
8	98	hsa-miR-202-3p	YES
9	98	hsa-miR-190a-3p	NO
10	98	hsa-miR-3130-5p	NO
11	98	hsa-miR-449b-5p	NO
12	97	hsa-miR-520h	NO
13	97	hsa-miR-335-3p	YES
14	97	hsa-miR-559	NO
15	97	hsa-miR-520g-3p	NO
16	97	hsa-miR-498-3p	NO
17	96	hsa-miR-1912-3p	NO
18	96	hsa-miR-302e	NO
19	96	hsa-miR-548ar-5p	NO
20	95	hsa-miR-372-3p	NO
21	95	hsa-miR-520e-3p	NO
22	95	hsa-miR-548j-5p	NO
23	95	hsa-miR-548c-5p	NO
24	95	hsa-miR-548b-5p	NO
25	95	hsa-miR-548ad-5p	NO
26	95	hsa-miR-520b-3p	NO
27	95	hsa-miR-520c-3p	NO
28	95	hsa-miR-548d-5p	NO
29	95	hsa-miR-548h-5p	NO
30	95	hsa-miR-373-3p	NO
31	95	hsa-miR-548a-5p	NO
32	95	hsa-miR-548i	NO
33	95	hsa-miR-548as-5p	NO
34	95	hsa-miR-548w	NO
35	95	hsa-miR-302c-3p	NO
36	95	hsa-miR-520d-3p	NO
37	95	hsa-miR-548ab	NO
38	95	hsa-miR-302a-3p	NO
39	95	hsa-miR-548ay-5p	NO
40	95	hsa-miR-548o-5p	NO
41	95	hsa-miR-520a-3p	NO
42	95	hsa-miR-548ap-5p	NO
43	95	hsa-miR-548au-5p	NO
44	95	hsa-miR-548y	NO
45	95	hsa-miR-302d-3p	NO
46	95	hsa-miR-302b-3p	NO
47	95	hsa-miR-548ae-5p	NO
48	95	hsa-miR-548am-5p	NO
49	95	hsa-miR-548aq-5p	NO
50	95	hsa-miR-548ak	NO
51	95	hsa-miR-548bb-5p	NO
52	95	hsa-miR-548l	NO
53	94	hsa-miR-4438	NO
54	94	hsa-miR-144-3p	YES
55	94	hsa-miR-4500	NO
56	94	hsa-miR-107	YES
57	94	hsa-miR-338-5p	NO
58	94	hsa-miR-103a-3p	NO
59	93	hsa-miR-1295b-5p	NO
60	93	hsa-miR-8068	NO

61	92	hsa-miR-383-3p	NO
62	92	hsa-miR-7-1-3p	NO
63	92	hsa-miR-548az-5p	NO
64	92	hsa-miR-548t-5p	NO
65	92	hsa-miR-29c-3p	YES
66	92	hsa-miR-7-2-3p	YES
67	92	hsa-miR-29a-3p	YES
68	92	hsa-miR-29b-3p	YES
69	91	hsa-miR-1250-3p	NO
70	91	hsa-miR-5590-3p	NO
71	91	hsa-miR-142-5p	NO
72	90	hsa-miR-765	NO
73	90	hsa-miR-505-3p	NO
74	89	hsa-let-7f-5p	NO
75	89	hsa-miR-98-5p	YES
76	89	hsa-let-7b-5p	NO
77	89	hsa-let-7g-5p	NO
78	89	hsa-miR-5681b	NO
79	89	hsa-let-7a-5p	NO
80	89	hsa-miR-582-5p	NO
81	89	hsa-let-7i-5p	NO
82	89	hsa-let-7e-5p	NO
83	89	hsa-let-7c-5p	NO
84	88	hsa-miR-551b-5p	NO
85	88	hsa-miR-5195-3p	NO
86	88	hsa-miR-4268	NO
87	87	hsa-miR-6852-3p	NO
88	87	hsa-miR-3910	NO
89	86	hsa-miR-502-3p	NO
90	86	hsa-miR-4458	NO
91	86	hsa-miR-501-3p	NO
92	86	hsa-let-7d-5p	NO
93	86	hsa-miR-145-5p	YES
94	85	hsa-miR-371a-5p	NO
95	84	hsa-miR-8054	NO
96	84	hsa-miR-513b-3p	NO
97	84	hsa-miR-548k	NO
98	84	hsa-miR-548av-5p	NO
99	83	hsa-miR-11181-3p	NO
100	83	hsa-miR-4456	NO
101	83	hsa-miR-5591-3p	NO
102	82	hsa-miR-19a-3p	YES
103	82	hsa-miR-19b-3p	YES
104	81	hsa-let-7a-3p	NO
105	81	hsa-miR-6069	NO
106	81	hsa-miR-98-3p	YES
107	81	hsa-miR-548c-3p	NO
108	81	hsa-let-7b-3p	NO
109	81	hsa-let-7f-1-3p	NO
110	80	hsa-miR-1277-5p	NO
111	80	hsa-miR-4678	NO
112	79	hsa-miR-4484	NO
113	78	hsa-miR-3909	NO
114	78	hsa-miR-888-5p	NO
115	78	hsa-miR-7151-3p	NO
116	77	hsa-miR-513a-3p	NO
117	77	hsa-miR-513c-3p	NO
118	76	hsa-miR-10398-5p	NO
119	76	hsa-miR-4319	NO
120	76	hsa-miR-381-3p	NO
121	76	hsa-miR-3606-3p	NO
122	76	hsa-miR-3609	NO
123	76	hsa-miR-300	NO

124	75	hsa-miR-1-5p	NO
125	74	hsa-miR-4698	NO
126	74	hsa-miR-5000-5p	NO
127	73	hsa-miR-877-3p	NO
128	73	hsa-miR-4709-3p	NO
129	72	hsa-miR-4486	NO
130	72	hsa-miR-3613-3p	NO
131	71	hsa-miR-6800-3p	NO
132	71	hsa-miR-6772-3p	NO
133	71	hsa-miR-6504-3p	NO
134	71	hsa-miR-4778-3p	NO
135	71	hsa-miR-3973	NO
136	71	hsa-miR-3924	NO
137	71	hsa-miR-10399-5p	NO
138	70	hsa-miR-6764-5p	NO
139	70	hsa-miR-338-3p	NO
140	69	hsa-miR-4666a-3p	NO
141	69	hsa-miR-548ah-5p	NO
142	68	hsa-miR-3692-5p	NO
143	67	hsa-miR-1915-3p	NO
144	67	hsa-miR-590-3p	NO
145	67	hsa-miR-6820-3p	NO
146	67	hsa-miR-5787	NO
147	67	hsa-miR-3164	NO
148	67	hsa-miR-138-5p	NO
149	67	hsa-miR-1323	NO
150	67	hsa-miR-125a-5p	NO
151	67	hsa-miR-125b-5p	NO
152	66	hsa-miR-6792-5p	NO
153	66	hsa-miR-539-3p	NO
154	66	hsa-miR-193a-3p	NO
155	66	hsa-miR-193b-3p	YES
156	66	hsa-miR-320e	NO
157	66	hsa-miR-485-3p	NO
158	65	hsa-miR-4277	NO
159	65	hsa-miR-500b-3p	NO
160	65	hsa-miR-6851-5p	NO
161	65	hsa-miR-3180-5p	NO
162	65	hsa-miR-452-3p	NO
163	65	hsa-miR-3689d	NO
164	64	hsa-miR-4503	NO
165	64	hsa-miR-7111-3p	NO
166	64	hsa-miR-32-3p	NO
167	63	hsa-miR-592	NO
168	63	hsa-miR-6771-3p	NO
169	63	hsa-miR-548o-3p	NO
170	63	hsa-miR-1199-3p	NO
171	62	hsa-miR-6836-5p	NO
172	62	hsa-miR-200c-3p	NO
173	62	hsa-miR-429	NO
174	62	hsa-miR-200b-3p	YES
175	61	hsa-miR-134-5p	NO
176	61	hsa-miR-4283	NO
177	61	hsa-miR-607	NO
178	61	hsa-miR-3118	NO
179	60	hsa-miR-518a-5p	NO
180	60	hsa-miR-527	NO
181	60	hsa-miR-4448	NO
182	60	hsa-miR-205-3p	NO
183	59	hsa-miR-510-3p	NO
184	59	hsa-miR-520d-5p	NO
185	59	hsa-miR-524-5p	NO
186	59	hsa-miR-3182	NO

187	58	hsa-miR-106b-5p	NO
188	58	hsa-miR-5707	NO
189	58	hsa-miR-526b-3p	NO
190	58	hsa-miR-4775	NO
191	58	hsa-miR-106a-5p	YES
192	58	hsa-miR-561-3p	NO
193	58	hsa-miR-20a-5p	YES
194	58	hsa-miR-4758-3p	NO
195	57	hsa-miR-4267	NO
196	56	hsa-miR-568	NO
197	56	hsa-miR-488-3p	NO
198	55	hsa-let-7f-2-3p	NO
199	55	hsa-miR-1185-1-3p	NO
200	55	hsa-miR-4761-5p	NO
201	55	hsa-miR-4505	NO
202	55	hsa-miR-331-5p	NO
203	55	hsa-miR-1185-2-3p	NO
204	54	hsa-miR-4756-3p	NO
205	54	hsa-miR-6751-5p	NO
206	54	hsa-miR-6894-3p	NO
207	53	hsa-miR-3185	NO
208	53	hsa-miR-6132	NO
209	53	hsa-miR-20b-5p	NO
210	53	hsa-miR-10522-5p	NO
211	53	hsa-miR-93-5p	NO
212	53	hsa-miR-17-5p	YES
213	53	hsa-miR-3168	NO
214	53	hsa-miR-153-5p	NO
215	53	hsa-miR-7159-3p	NO
216	53	hsa-miR-519d-3p	NO
217	52	hsa-miR-515-5p	NO
218	52	hsa-miR-519e-5p	NO
219	52	hsa-miR-4796-3p	NO
220	51	hsa-miR-3144-5p	NO
221	51	hsa-miR-626	NO
222	51	hsa-miR-890	NO
223	51	hsa-miR-320a-5p	NO
224	51	hsa-miR-4652-3p	NO
225	51	hsa-miR-6876-3p	NO
226	51	hsa-miR-6131	NO
227	50	hsa-miR-6783-5p	NO
228	50	hsa-miR-5193	NO
229	50	hsa-miR-6512-3p	NO
230	50	hsa-miR-766-3p	NO
231	50	hsa-miR-6770-3p	NO
232	50	hsa-miR-4699-3p	NO
233	50	hsa-miR-8063	NO
234	50	hsa-miR-6720-5p	NO

Table S1. Predicted miRNAs as potential targets for the *MYCN* gene. miRNAs were predicted using the miRDB database (107-110).
